# Shared gene signatures and biochemical regulatory networks linking Parkinson’s disease and ulcerative colitis

**DOI:** 10.1038/s41531-026-01374-z

**Published:** 2026-05-06

**Authors:** Xiaohui Sun, Zhichen An, Shufei Wang, Kongjia Wang

**Affiliations:** 1 Department of Neurology, Beijing Tsinghua Changgung Hospital, School of Clinical Medicine, Tsinghua Medicine, Tsinghua University, Beijing, China; 2 IDG/McGovern Institute for Brain Research at Tsinghua University, Tsinghua University, Beijing, China; 3 Orthopaedics and Sports Medicine Center, Beijing Tsinghua Changgung Hospital, School of Clinical Medicine, Tsinghua Medicine, Tsinghua University, Beijing, China; 4 College of Clinical and Basic Medical Sciences, Shandong First Medical University & Shandong Academy of Medical Sciences, Jinan, China; 5 Clinical Research Centre (CRC), Department of Clinical Sciences, Lund University, Malmö, Sweden

**Keywords:** Biomarkers, Computational biology and bioinformatics, Diseases, Immunology

## Abstract

Epidemiological studies suggest an association between Parkinson’s disease (PD) and ulcerative colitis (UC), yet the molecular programs potentially linking these disorders remain poorly defined. Here, we integrated curated disease-gene resources with publicly available blood transcriptomic datasets to identify shared molecular features across PD and UC. We identified 320 shared signature genes and a topology-derived 10-gene core module that included *TNF,* *IL1B,* *TP53*, *BCL2*, and *CASP3*. Enrichment analyses implicated a convergent inflammatory-stress architecture characterized by microbial-response, oxidative-stress, lipid/inflammatory, and IL-17-related signaling programs. Immune deconvolution revealed partially overlapping peripheral immune alterations in PD and UC, most notably reduced memory B-cell abundance. Network analyses further highlighted TP53 and JUN as major transcriptional hubs and prioritized several candidate compounds for follow-up investigation. Cross-validation and external validation showed that only a subset of the core genes retained stable discriminatory performance across cohorts, indicating that network centrality did not uniformly translate into robust classifier-like behavior. Collectively, these findings support a shared inflammatory/apoptotic regulatory module linking PD and UC and provide a systems-level framework for mechanistic studies and therapeutic prioritization, rather than a definitive biomarker panel.

## Introduction

The gut–brain axis has emerged as a key framework for understanding bidirectional interactions between neurological and gastrointestinal disorders, particularly Parkinson’s disease (PD) and ulcerative colitis (UC)^1–5^. UC, a chronic inflammatory bowel disease characterized by immune dysregulation and microbial dysbiosis, shares mechanistic features with inflammatory and stress-response pathways implicated in PD^6,7^. Epidemiological studies further support a link between the two conditions, suggesting bidirectional comorbidity^1,8^. Moreover, the detection of α-synuclein pathology within the enteric nervous system lends biological plausibility to gut-first models of PD, in which pathology may originate in the gastrointestinal tract before propagating to the central nervous system^6,9,10^.

Despite this clinical and biological convergence, the shared molecular architecture linking PD and UC remains poorly defined, particularly with respect to immune and metabolic regulatory pathways. Most previous studies have examined these disorders in isolation, leaving their common molecular programs insufficiently resolved. Here, we integrated multi-source disease-gene resources with public transcriptomic datasets to identify shared gene signatures, regulatory circuits, and candidate therapeutic targets across PD and UC. By delineating these convergent molecular features, we aimed to provide a systems-level framework for mechanistic investigation and therapeutic prioritization within the gut–brain axis.

## Results

### Integrated identification of shared signature genes

**Fig. 1:**
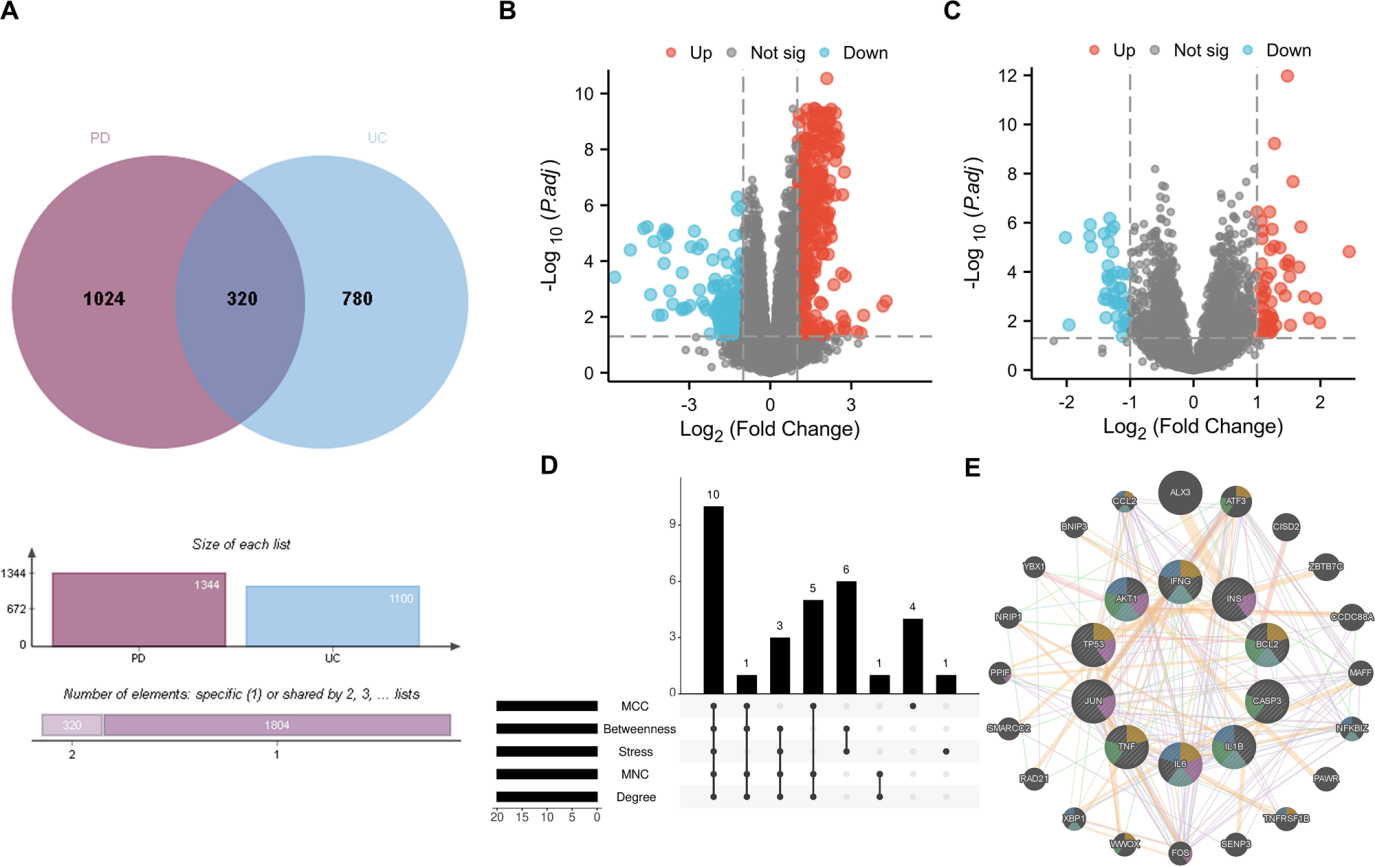
Identification of shared signature genes between Parkinson’s disease (PD) and ulcerative colitis (UC). **A** Identification of shared signatures: The Venn diagram illustrates the intersection of disease-associated gene sets from multiple sources. A total of 320 shared signature genes were identified by integrating transcriptomic DEGs from GEO datasets (GSE22491 for PD and GSE3365 for UC, applying a stringent threshold of FDR < 1e-5 and |log₂FC| > 1), with curated disease-associated genes from GeneCards, DisGeNET, and CTD databases. Transcriptomic profiles: Volcano plots display the gene expression landscape in **B** PD (GSE22491) and **C** UC (GSE3365). Red and blue dots represent significantly up-regulated and down-regulated genes, respectively, based on the predefined empirical Bayes (eBayes) moderation method. **D** Consensus of core genes: The Upset plot visualizes the consensus selection of the 10 core genes (*TNF, IL1B, TP53, AKT1, CASP3, IL6, BCL2, IFNG, INS,* and *JUN*). These genes represent the consensus candidates identified across five distinct topological algorithms (Degree, MCC, MNC, Stress, Betweenness) using the CytoHubba plugin within the protein-protein interaction (PPI) network. **E** GeneMANIA interaction network of core genes: A functional interaction network was constructed specifically for the 10 core genes to explore their biological interplay. The network integrates multiple evidence levels: co-expression (59.0%), physical interactions (15.2%), and predicted functional associations (22.1%), highlighting the highly interconnected nature of these neuro-inflammatory and apoptotic regulators.

**Fig. 2:**
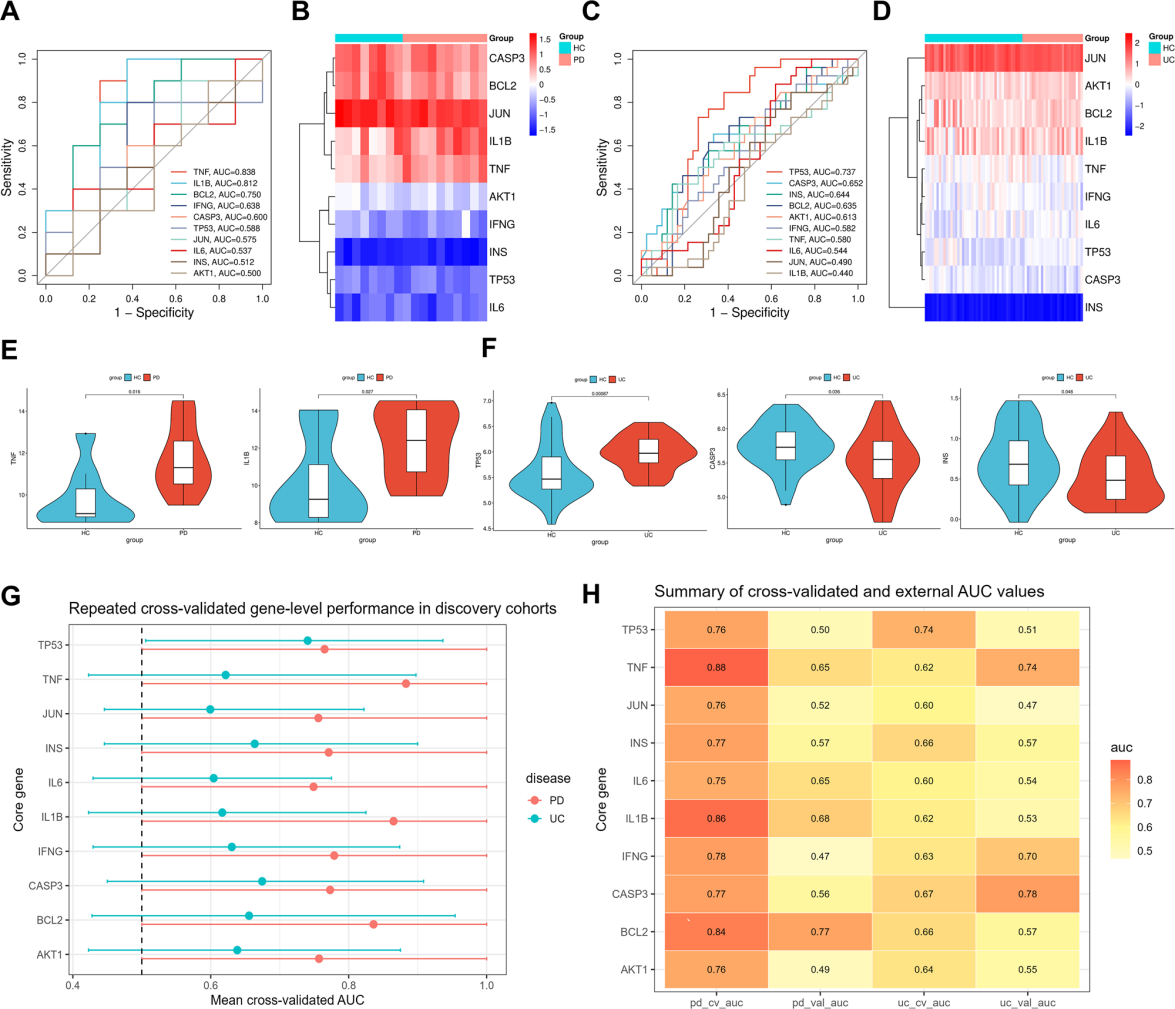
Expression patterns and gene-level discriminatory performance of the 10 topology-derived core genes in PD and UC cohorts. Receiver operating characteristic (ROC) curves evaluating the discriminatory capacity of the ten core genes in **A** PD (GSE22491) and **C** UC (GSE3365) discovery cohorts. The area under the curve (AUC) values provide a descriptive estimate of gene-level discriminatory performance to distinguish disease states from healthy controls. Hierarchical clustering heatmaps of the ten core genes in **B** PD and **D** UC samples. Samples are categorized by color (Red: Disease; Blue: Healthy Controls). Clustering was implemented using the complete linkage method based on Euclidean distance metrics to illustrate coordinated expression patterns across groups. Box plots illustrating the differential expression levels of representative core genes in (**E**) PD and (**F**) UC. *P*-values were determined by the non-parametric Wilcoxon rank-sum test, with *P* < 0.05 considered as the threshold for statistical significance. Data are presented as medians with interquartile ranges (IQR). All statistical tests were two-tailed. **G** Repeated stratified 5-fold cross-validated area under the curve (AUC) values for each topology-derived core gene in the discovery cohorts of Parkinson’s disease (PD) and ulcerative colitis (UC). Points indicate mean AUC values across repeated iterations, and error bars indicate empirical 95% intervals derived from the cross-validation procedure. A dashed horizontal line marks the chance level (AUC = 0.5). **H** Heatmap-style summary of cross-validated and external validation AUC values for all topology-derived core genes across the PD and UC analysis framework. Numeric values are shown within each tile to facilitate direct comparison. These analyses were conducted separately in PD and UC to assess gene-level stability and reproducibility across cohorts. Overall, the topology-derived core genes showed heterogeneous discriminatory performance, with a narrower subset demonstrating relatively greater stability, whereas several others showed context-dependent or limited standalone discriminatory ability.

To establish a molecular foundation for the PD-UC axis, we integrated multi-source genomic and transcriptomic data. Integration of disease-associated genes from curated databases and differentially expressed genes (DEGs, Supplementary Data 1) from our transcriptomic analysis identified 320 shared signature genes (Table 1, Fig. 1A, and Supplementary Data 2). During the transcriptomic discovery phase, a stringent statistical refinement was employed to prioritize genes with the highest biological relevance and to minimize false discovery rates. Under the threshold of FDR < 0.00001 and |log_2_FC| > 1, 185 DEGs were identified in the PD discovery cohort (GSE22491), while 20 genes met these criteria in the UC cohort (GSE3365) (Fig. 2B, C). These shared signature genes constitute a potential candidate set for exploring the shared molecular features linking the two disorders.

**Table 1:** Integration of multi-source genomic and transcriptomic data to identify shared signature genes between PD and UC

Disease	Data type	Data source	Raw number	Filter condition	After filtering	Merge	Common
PD	Database	GeneCards	9145	If the raw data are more than 500, the top 500 are included	500	1344	320
		DisGeNET	2078		500		
		CTD	29347		500		
	GEO	GSE22491	9990	*P*_FDR_ < 0.00001 and |log_2_FC| > 1	185		
UC	Database	GeneCards	5401	If the raw data are more than 500, the top 500 are included	500	1100	
		DisGeNET	1458		500		
		CTD	29051		500		
	GEO	GSE3365	12548	*P*_FDR_ < 0.00001 and |log_2_FC| > 1	20		

To maintain computational stringency and focus on the most relevant genetic associations, the top 500 genes (ranked by relevance scores) were extracted from GeneCards, DisGeNET, and CTD databases when the initial retrieval exceeded this threshold. The “Merge” column represents the unique union of genes identified across multiple databases and transcriptomic datasets for each disease, while “Common” indicates the final intersection of signature genes shared between PD and UC.

*PD* Parkinson’s disease, *UC* ulcerative colitis, *CTD* Comparative Toxicogenomics Database, *GEO* Gene Expression Omnibus, *GSE* Gene Expression Omnibus Series, *PFDR* permutation-based false discovery rate, *log*_2_*FC* log_2_ fold-change.

### PPI network architecture and core gene identification

To define the shared protein-interaction landscape between PD and UC, we constructed a protein-protein interaction (PPI) network from the 320 shared signature genes. To minimize the influence of ubiquitous, non-specific hubs, housekeeping-associated proteins, including glyceraldehyde-3-phosphate dehydrogenase (*GAPDH*), actin beta (*ACTB*), and albumin (ALB), were excluded before topological analysis. Integration of five complementary centrality measures—MCC, Stress, MNC, Degree, and Betweenness—identified 10 candidate hub genes that consistently ranked among the top 20 across all algorithms. These topology-derived core genes were *BCL2, INS, IL1B, JUN, TNF, TP53, IFNG, CASP3, IL6*, and *AKT1* (Table 2 and Supplementary Table 2).

**Table 2:** Topological importance and discriminatory performance of the core genes across cohorts

Gene symbol	MCC rank	Stress rank	MNC rank	Degree rank	Betweenness rank	PD GSE22491 (AUC)	PD GSE75249 (AUC)	UC GSE3365(AUC)	UC GSE119600 (AUC)
AKT1	2	4	2	2	1	0.500	0.489	0.613	0.545
IL6	1	2	3	3	4	0.537	0.648	0.544	0.545
TNF	19	1	1	1	3	0.838	0.654	0.580	0.745
IL1B	6	5	5	5	6	0.812	0.684	0.440	0.526
TP53	20	3	4	4	2	0.588	0.503	0.737	0.514
INS	15	6	6	6	5	0.512	0.566	0.644	0.570
CASP3	7	15	7	7	15	0.600	0.560	0.652	0.778
JUN	18	10	9	9	11	0.575	0.522	0.490	0.474
BCL2	5	19	8	8	18	0.750	0.766	0.635	0.568
IFNG	12	17	14	14	14	0.638	0.467	0.582	0.697

*MCC* maximal clique centrality, *MNC* maximum neighborhood component, *PD* Parkinson’s disease, *UC* ulcerative colitis, *GSE* Gene Expression Omnibus Series, *AUC* area under the receiver operating characteristic curve, *AKT1* AKT serine/threonine kinase 1, *IL6* interleukin 6, *TNF* tumor necrosis factor, *IL1B* interleukin 1 beta, *TP53* tumor protein P53, *INS* insulin, *CASP3* caspase 3, *JUN* Jun proto-oncogene, *BCL2* B-cell lymphoma 2, *IFNG* interferon gamma.

We next evaluated the gene-level discriminatory performance of these topology-derived core genes in the PD and UC cohorts using receiver operating characteristic (ROC) analysis (Table 2). In the PD discovery cohort (GSE22491), *TNF* (AUC = 0.838) and *IL1B* (AUC = 0.812) showed relatively higher gene-level discriminatory performance than the other topology-derived core genes (Fig. 2A), with partial support in the external PD validation cohort (GSE75249; *TNF* AUC = 0.654, *IL1B* AUC = 0.684). In the UC analysis framework, *CASP3* exhibited the most consistent performance across the discovery cohort (GSE3365; AUC = 0.652) and the external validation cohort (GSE119600; AUC = 0.778; Fig. 2C). Differential expression analysis further supported disease-associated perturbation of the shared module: *TNF* and *IL1B* were significantly upregulated in the PD discovery cohort (*P* < 0.05; Fig. 2E), whereas *TP53, CASP3*, and *INS* were significantly differentially expressed in the UC discovery cohort (*P* < 0.05; Fig. 2F). Together, these analyses were intended to characterize the expression patterns and descriptive discriminatory capacity of the topology-derived core genes, rather than to establish a definitive diagnostic signature. Additional ROC curves, hierarchical clustering, and expression profiles across discovery and validation cohorts are provided in Supplementary Fig. 1.

Notably, the discriminatory performance of the 10 topology-derived core genes was heterogeneous across datasets. Cross-validated analyses further showed that not all network-central genes retained stable single-gene performance across cohorts, and that the more reproducible signal was concentrated in a narrower subset of inflammatory and apoptosis-related regulators. These findings indicate that network centrality and gene-level classification performance capture related, but distinct, properties of the shared PD-UC molecular architecture. Accordingly, the 10-gene core set is better interpreted as a topology-informed shared regulatory module linking PD and UC than as a finalized biomarker panel (Fig. 2H; Table 3 and Supplementary Fig. 4).

**Table 3:** Cross-validated and external validation performance of core genes

	PD		UC		
Gene	CV AUC (95% CI)	Val. AUC (95% CI)	CV AUC (95%CI)	Val. AUC (95% CI)	Note
*TNF*	0.883 (0.500–1.000)	0.654 (0.442–0.866)	0.622 (0.423–0.898)	0.745 (0.663–0.826)	Strong support
*CASP3*	0.773 (0.500–1.000)	0.560 (0.328–0.793)	0.675 (0.450–0.909)	0.778 (0.697–0.859)	Strong support
*BCL2*	0.836 (0.500–1.000)	0.766 (0.567–0.966)	0.656 (0.428–0.954)	0.568 (0.471–0.666)	Strong support
*TP53*	0.765 (0.500–1.000)	0.503 (0.270–0.735)	0.740 (0.506–0.937)	0.514 (0.416–0.613)	Context-dep.
*AKT1*	0.757 (0.500–1.000)	0.489 (0.258–0.720)	0.638 (0.423–0.875)	0.545 (0.451–0.640)	Context-dep.
*IFNG*	0.779 (0.500–1.000)	0.467 (0.235–0.699)	0.630 (0.429–0.874)	0.697 (0.607–0.787)	Context-dep.
*JUN*	0.756 (0.500–1.000)	0.522 (0.291–0.753)	0.599 (0.446–0.822)	0.474 (0.378–0.571)	Context-dep.
*INS*	0.771 (0.500–1.000)	0.566 (0.336–0.796)	0.664 (0.446–0.900)	0.570 (0.470–0.671)	Context-dep.
*IL1B*	0.865 (0.500–1.000)	0.684 (0.477–0.891)	0.617 (0.423–0.825)	0.526 (0.424–0.627)	Ltd. Repro.
*IL6*	0.749 (0.500–1.000)	0.648 (0.426–0.871)	0.604 (0.429–0.775)	0.545 (0.439–0.651)	Ltd. Repro.

Values represent mean AUC with 95% CIs in parentheses. CV AUC was calculated via repeated stratified 5-fold cross-validation in discovery cohorts (GSE22491, PD; GSE3365, UC), while Val. AUC was estimated in independent cohorts (GSE75249, PD; GSE119600, UC). Core genes were grouped descriptively into three categories (Strong support, Context-dep., or Ltd. Repro.) according to their overall stability across internal cross-validation and external validation, with the aim of distinguishing topological centrality from reproducible discriminatory performance.

*PD* Parkinson’s disease, *UC* ulcerative colitis, *AUC* area under the receiver operating characteristic curve, *CV* cross-validation, *Val.* external validation, *CI* confidence interval, *Context-dep.* context-dependent, *Ltd. Repro.* limited standalone reproducibility, *AKT1* AKT serine/threonine kinase 1, *IL6* interleukin 6, *TNF* tumor necrosis factor, *IL1B* interleukin 1 beta, *TP53* tumor protein P53, *INS* insulin, *CASP3* caspase 3, *JUN* Jun proto-oncogene, *BCL2* B-cell lymphoma 2, *IFNG* interferon gamma.

### Functional enrichment and cross-cohort comparator analysis

**Fig. 3:**
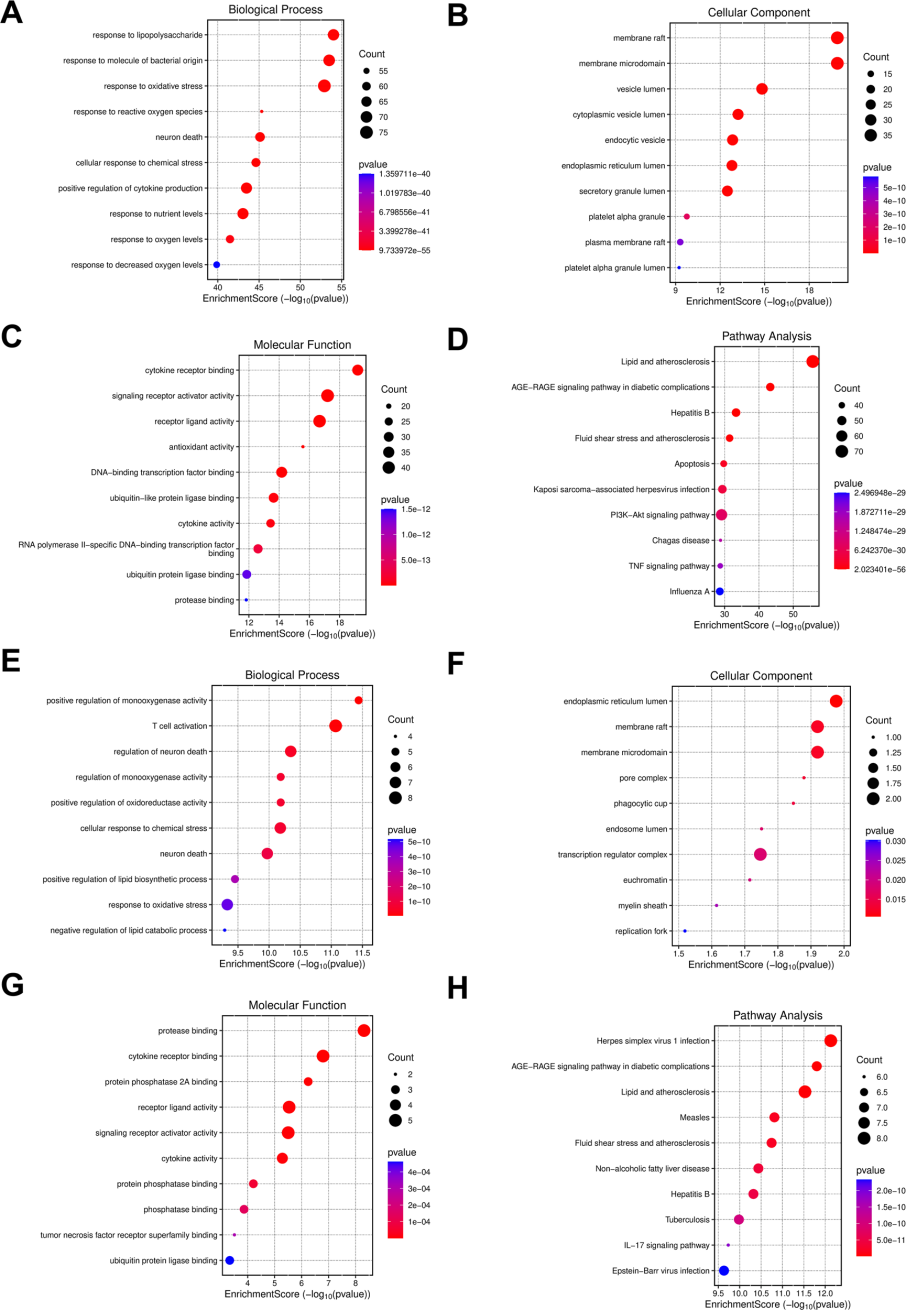
Functional enrichment landscape of shared signature genes and core candidates. Gene Ontology (GO) enrichment analysis of the 320 shared signature genes, stratified into **A** biological process (BP), **B** cellular component (CC), and **C** molecular function (MF). The results highlight a broader inflammatory-stress landscape involving microbial-response programs, oxidative stress, cytokine-related activity, and lipid/inflammatory pathway enrichment. **D** Kyoto Encyclopedia of Genes and Genomes (KEGG) pathway analysis for shared signature genes, identifying key metabolic and inflammatory pathways linking PD and UC. GO term enrichment for the ten identified core genes (**E** BP, **F** CC, **G** MF), showing a high concentration in programmed cell death and stress-activated pathways. **H** KEGG enrichment analysis of the 10 core genes. Bubble size corresponds to the gene count, while the color gradient (blue to red) represents statistical significance (adjusted *P*-value). All terms were filtered using a Benjamini–Hochberg adjusted *P* < 0.05.

Functional enrichment analysis of the 320 shared signature genes revealed a broad inflammatory-stress landscape characterized by microbial-response, oxidative-stress, and lipid-associated programs (Fig. 3 and Table 4). Within the GO biological process category, the most significantly enriched terms included response to lipopolysaccharide (*P*_adj_ = 4.94 × 10^−51^) and response to oxidative stress (*P*_adj_ = 2.08 × 10^−50^), while molecular function analysis highlighted cytokine receptor binding (*P*_adj_ = 4.44 × 10^−17^). KEGG pathway analysis identified lipid and atherosclerosis (*P*_adj_ = 5.78 × 10^−54^) and the IL-17 signaling pathway (*P*_adj_ = 2.11 × 10^−26^) among the most enriched pathways, supporting the involvement of inflammatory and lipid-associated regulatory programs in the shared PD-UC transcriptomic landscape.

**Table 4:** Functional landscape of shared signature and core genes in PD and UC

Gene group	Category	Term ID	Description	Count	Adjusted *P*-value	Representative genes
Shared (*n* = 320)	KEGG	hsa05417	Lipid and atherosclerosis	71	5.79E-54	AKT1, TNF, IL6, INS, TP53
	KEGG	hsa04657	IL-17 signaling pathway	33	2.11E-26	TNF, IL6, CXCL8, CASP3
	GO:BP	GO:0032496	Response to lipopolysaccharide	69	4.94E-51	TLR4, CD14, TNF, IL6
	GO:BP	GO:0002237	Response to molecule of bacterial origin	70	8.28E-51	TLR4, CD14, TNF, IL6, NOD2
	GO:BP	GO:0006979	Response to oxidative stress	75	2.08E-50	HMOX1, SOD2, CAT, AKT1
	GO:CC	GO:0045121	Membrane raft	37	2.46E-18	CD14, TLR4, LRRK2, LYN
	GO:CC	GO:0034774	Secretory granule lumen	47	3.51E-17	IL6, TNF, CCL5, CXCL8
	GO:MF	GO:0005126	Cytokine receptor binding	34	4.45E-17	TNFRSF1A, IL6R, IL1R1
Core (*n* = 10)	KEGG	hsa05417	Lipid and atherosclerosis	8	< lower numerical limit*	IL6, AKT1, BCL2, IL1B, CASP3
	KEGG	hsa04657	IL-17 signaling pathway	6	< lower numerical limit*	IL6, IL1B, CASP3, IFNG, TNF
	GO:BP	GO:0042110	T cell activation	8	< lower numerical limit*	IL6, AKT1, BCL2, IL1B, CASP3
	GO:BP	GO:1901214	Regulation of neuron death	7	< lower numerical limit*	AKT1, BCL2, CASP3, JUN, TNF
	GO:BP	GO:0006979	Response to oxidative stress	7	< lower numerical limit*	IL6, AKT1, BCL2, CASP3, TP53
	GO:MF	GO:0002020	Protease binding	5	< lower numerical limit*	BCL2, CASP3, INS, TNF, TP53
	GO:MF	GO:0005126	Cytokine receptor binding	5	8.0E-06	IL6, IL1B, CASP3, IFNG, TNF

Adjusted *P*-values were corrected using the Benjamini–Hochberg procedure. Count denotes the number of overlapping genes in each enriched term. For several enrichment terms in the 10-gene core set, the exported adjusted *P*-values were displayed as 0 because of numerical underflow; these should be interpreted as extremely small adjusted *P*-values rather than true zeros (indicated by <lower numerical limit*). No cellular component term for the core-gene set passed the FDR threshold and was therefore not prioritized in the main table.

*FDR* false discovery rate, *GO* Gene Ontology, *KEGG* Kyoto Encyclopedia of Genes and Genomes, *BP* biological process, *CC* cellular component, *MF* molecular function.

Enrichment analysis of the 10 topology-derived core genes revealed a narrower, but partially overlapping, functional profile dominated by immune-, apoptosis-, and stress-related processes, including T-cell activation, regulation of neuron death, response to oxidative stress, protease binding, and cytokine receptor binding. At the pathway level, this reduced gene set remained broadly consistent with the larger shared-gene signature, particularly with respect to inflammatory and lipid-associated signaling, but represented a more focused regulatory module rather than the full functional breadth of the 320-gene landscape. Sensitivity analysis using relaxed statistical thresholds yielded a broadly similar shared-signature overlap and core topological architecture, supporting the robustness of the principal findings (Supplementary Fig. 3).

To place these enrichment patterns in a cross-disease context, we additionally analyzed two blood-based AD cohorts. These comparator analyses were not intended as formal validation, but rather to assess whether the inflammatory/lipid-associated enrichment observed in the shared PD-UC signature would also be recapitulated in an independent disease setting with a distinct neuroimmune background. In contrast to the PD-UC signature, the AD comparator cohorts did not show a reproducible inflammatory/lipid-associated enrichment pattern. In GSE97760, the dominant signals were related to proteasome-mediated ubiquitin-dependent protein catabolic processes, protein quality-control pathways, and RNA splicing, whereas GSE63060 was enriched mainly for translation, ribosome-related, and electron transport/oxidative phosphorylation pathways (Supplementary Table 5). Accordingly, the AD comparator results neither reproducibly recapitulated the inflammatory/lipid-associated enrichment pattern observed in the PD-UC signature nor converged with each other. Disease-named neurodegeneration pathways identified in GSE63060 were therefore interpreted cautiously, as they most likely reflected broader mitochondrial or proteostasis-related programs rather than PD-specific recapitulation.

To further interrogate the IL-17 signal identified in the discovery analysis, we performed independent cohort-level enrichment analysis using a fixed KEGG IL-17 gene set. Significant enrichment was observed in both the UC discovery and validation cohorts, supporting reproducible pathway involvement in UC. By contrast, the PD cohorts showed positive but non-significant enrichment scores, whereas the two AD comparator cohorts were both non-significant and directionally inconsistent (Supplementary Fig. 5 and Supplementary Table 4). Collectively, these findings support IL-17 signaling as a biologically plausible component of the shared PD-UC inflammatory landscape, with stronger independent support in UC than in PD. More broadly, the shared PD-UC transcriptomic profile was characterized by microbial-response, oxidative-stress, and lipid/inflammatory programs, whereas the AD comparator results should be interpreted as exploratory contextual evidence rather than as a definitive test of disease specificity. Importantly, pathway-level enrichment does not imply that every individual IL-17 pathway member has strong standalone discriminatory performance. Rather, the enrichment signal likely reflects coordinated perturbation across multiple pathway components, which can coexist with weak or context-dependent AUC values for individual genes, including *IL6*.

### Characterization of the immune microenvironment

**Fig. 4:**
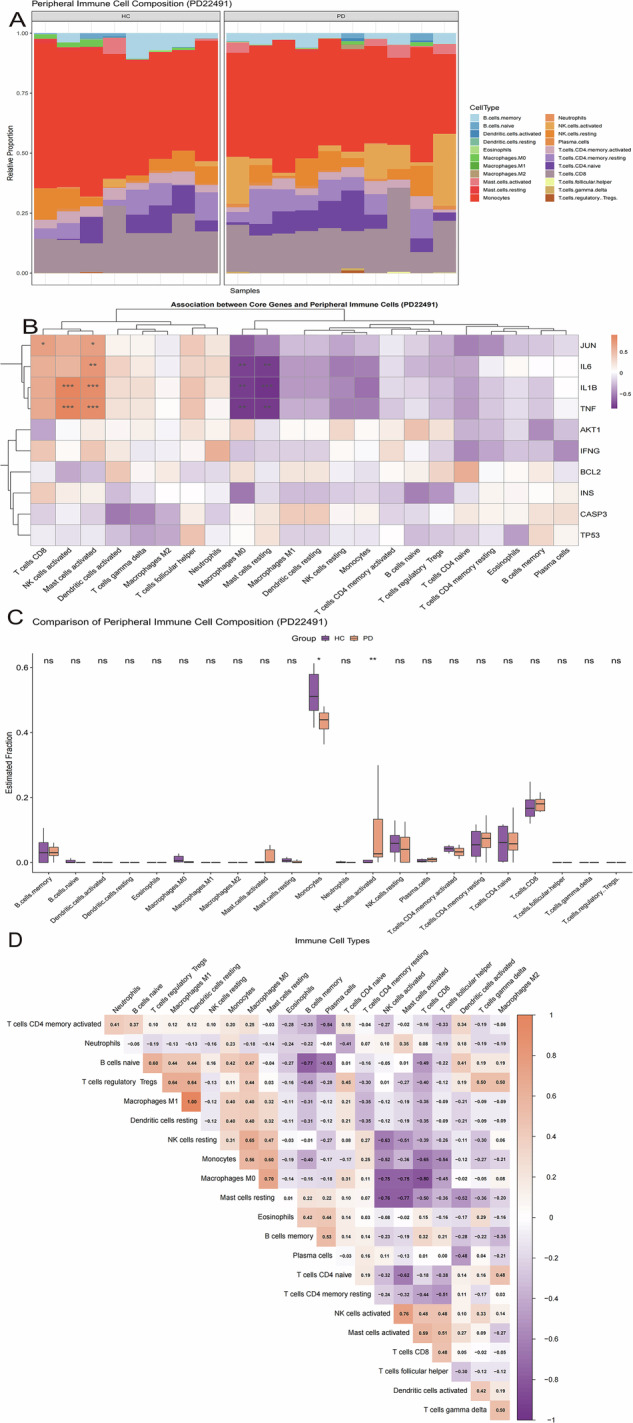
Peripheral immune cell composition and its association with core genes in Parkinson’s disease. **A** Relative proportions of 22 human leukocyte subsets (LM22) estimated by CIBERSORTx in the PD dataset (GSE22491). Each bar represents one sample, and each color corresponds to a distinct immune cell type. **B** Spearman correlation heatmap showing the associations between the 10 topology-derived core genes and immune cell subsets in PD. Red indicates positive correlation and blue indicates negative correlation. **C** Comparison of immune cell fractions between PD and healthy controls (HC). Statistical significance was assessed using the Wilcoxon rank-sum test. **D** Correlation matrix showing the internal relationships among the 22 immune cell types in PD, illustrating the coordinated peripheral immune microenvironment. For panels involving statistical comparisons or correlation analyses, *P*-values were adjusted using the Benjamini–Hochberg (BH) method.

**Fig. 5:**
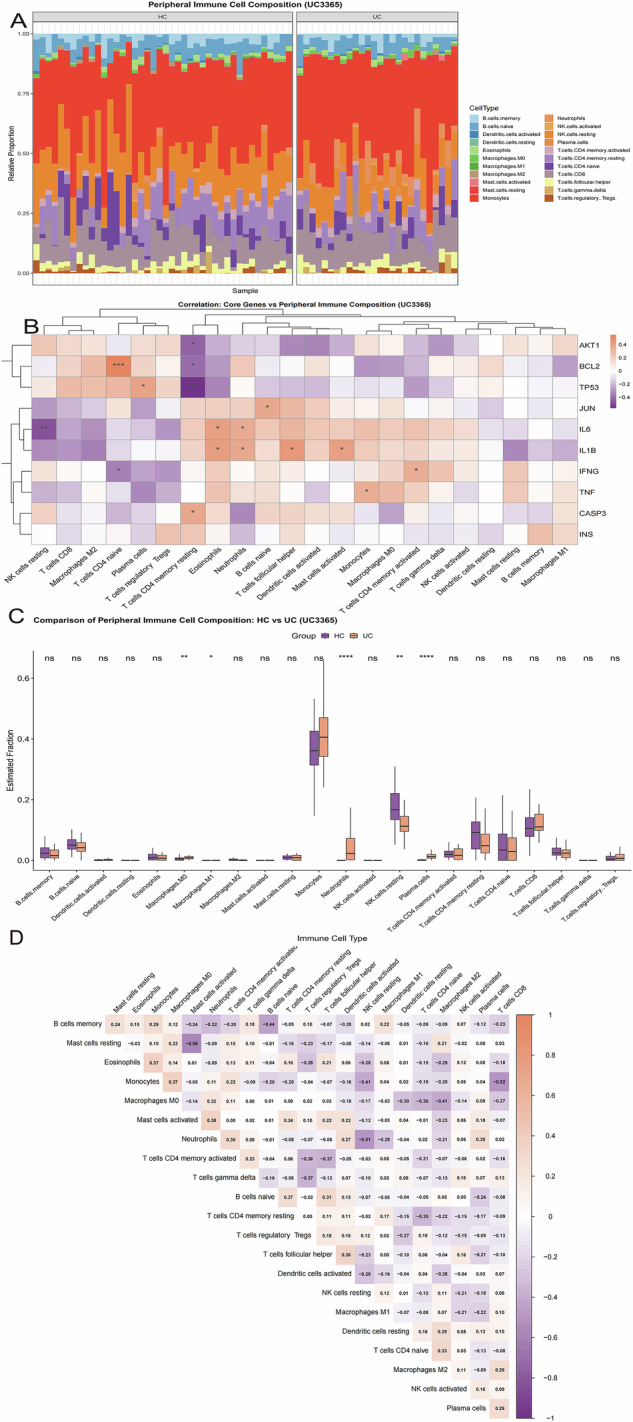
Peripheral immune cell composition and its association with core genes in ulcerative colitis. **A** Relative proportions of 22 human leukocyte subsets (LM22) estimated by CIBERSORTx in the UC dataset (GSE3365). Each bar represents one sample, and each color corresponds to a distinct immune cell type. **B** Spearman correlation heatmap showing the associations between the 10 topology-derived core genes and immune cell subsets in UC. Red indicates positive correlation and blue indicates negative correlation. **C** Comparison of immune cell fractions between UC and healthy controls (HC). Statistical significance was assessed using the Wilcoxon rank-sum test. **D** Correlation matrix showing the internal relationships among the 22 immune cell types in UC, illustrating the coordinated peripheral immune microenvironment. All *P*-values were adjusted using the Benjamini–Hochberg (BH) method.

CIBERSORTx was used to estimate the relative proportions of 22 immune cell subsets in the discovery cohorts (Figs. 4A and 5A). In PD (GSE22491), monocyte proportions were significantly reduced, whereas activated NK-cell proportions were significantly increased (*P* < 0.05 and *P* < 0.01, respectively; Fig. 4C). A broadly similar pattern was observed in the independent PD validation cohort (Supplementary Fig. 2). In UC (GSE3365), a more extensive inflammatory profile was evident, characterized by increased neutrophil, monocyte, and regulatory T-cell (Treg) proportions together with reduced memory B-cell and M2 macrophage proportions (all *P* < 0.05; Fig. 5C). Notably, reduced memory B-cell abundance was observed in both disease settings, consistent with a potentially shared feature of peripheral immune dysregulation.

Spearman correlation analysis was then performed to assess associations between the 10 topology-derived core genes and immune-cell proportions (Figs. 4B and 5B). In PD, *TNF* and *IL1B* were strongly positively correlated with activated NK cells and activated mast cells (*R* > 0.8, *P* < 0.001), whereas *JUN* was positively associated with CD8^+^ T cell abundance (*R* = 0.69, *P* < 0.01; Fig. 4B). In UC, partially concordant patterns were observed, with *TNF, IL1B*, and *IL6* positively correlated with neutrophils and activated dendritic cells (Fig. 5B). Together, these results indicate that inflammatory core genes are linked to distinct yet partially overlapping immune alterations in PD and UC.

Correlation matrices of immune-cell subsets further revealed coordinated relationships within each disease context (Figs. 4D and 5D). In both PD and UC, resting and activated states of related immune-cell populations tended to be negatively correlated, consistent with shifts in immune activation states. In addition, monocytes showed negative correlations with several lymphoid subsets across cohorts, supporting a peripheral immune imbalance characterized by relative myeloid predominance and lymphoid dysregulation. Complete immune-cell proportion profiles, group comparisons, and gene-immune correlation results for the discovery and validation cohorts are provided in Supplementary Fig. 2 and Supplementary Data 4.

### Gene regulatory network construction

**Fig. 6:**
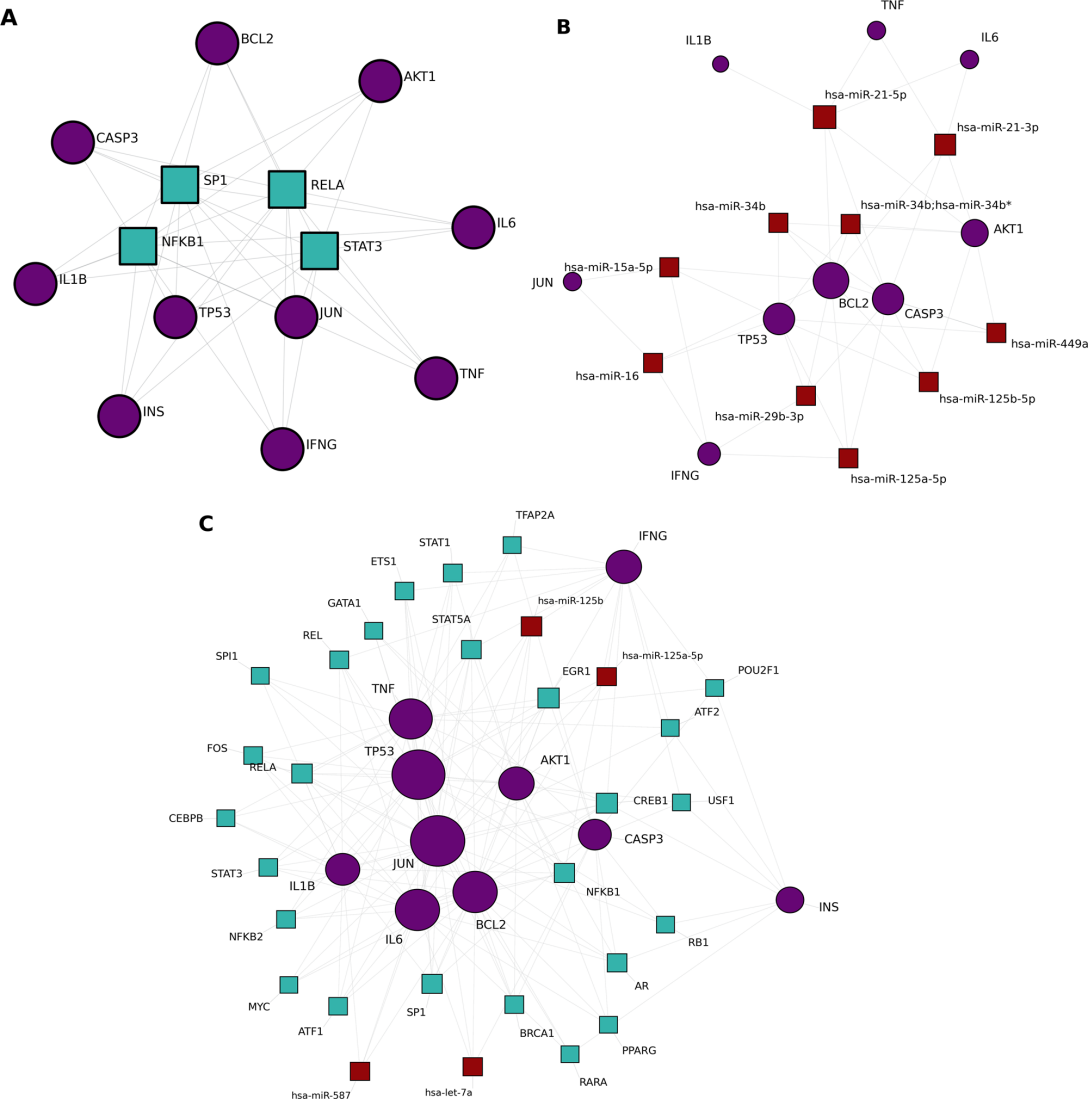
Transcriptional and post-transcriptional regulatory architecture of PD–UC core genes. **A** Transcription factor (TF)–gene interaction network. Squares represent upstream TFs and circles represent the identified core genes as predicted via the OmniPath database. *TP53* and *JUN* emerge as the primary regulatory hubs coordinating the transcriptional landscape across PD and UC. **B** MicroRNA (miRNA)–gene interaction network. Integrated mapping of miRNAs targeting the ten core genes. hsa-miR-21-5p demonstrates significant regulatory breadth, suggesting its role as a potential post-transcriptional modulator within the gut-brain axis. **C** Integrated TF–miRNA regulatory circuit. A synergistic regulatory landscape of the core genes was established using RegNetwork to illustrate coordinated control at both transcriptional and post-transcriptional levels. All networks were filtered using a degree-based strategy (Degree > 9 for TFs, Degree > 3 for miRNAs) to prioritize high-confidence regulatory interactions and minimize topological noise.

The TF-gene regulatory network comprised 61 nodes representing predicted transcriptional regulators and the 10 topology-derived core genes (Fig. 6A and Supplementary Data 5). Among the predicted transcription factors (TFs), TP53 (degree = 59) and JUN (degree = 58) emerged as the highest-degree regulatory hubs. Other prominent TFs included NFKB1, RELA, and STAT3, consistent with a coordinated transcriptional program centered on cellular stress and inflammatory signaling. Complementary miRNA-gene network analysis (Fig. 6B) identified *BCL2* and *CASP3* as the most heavily targeted nodes at the post-transcriptional level. Using the same topological framework, hsa-miR-21-5p and hsa-miR-21-3p emerged as the leading miRNA regulators (Supplementary Data 5). Integration of the TF, miRNA, and core-gene layers (Fig. 6C) revealed a densely interconnected regulatory architecture with potential feed-forward and feedback relationships, supporting the view that these core genes participate in a coordinated disease-associated regulatory program shared by PD and UC.

### Integrated regulatory networks and therapeutic associations in PD and UC

**Fig. 7:**
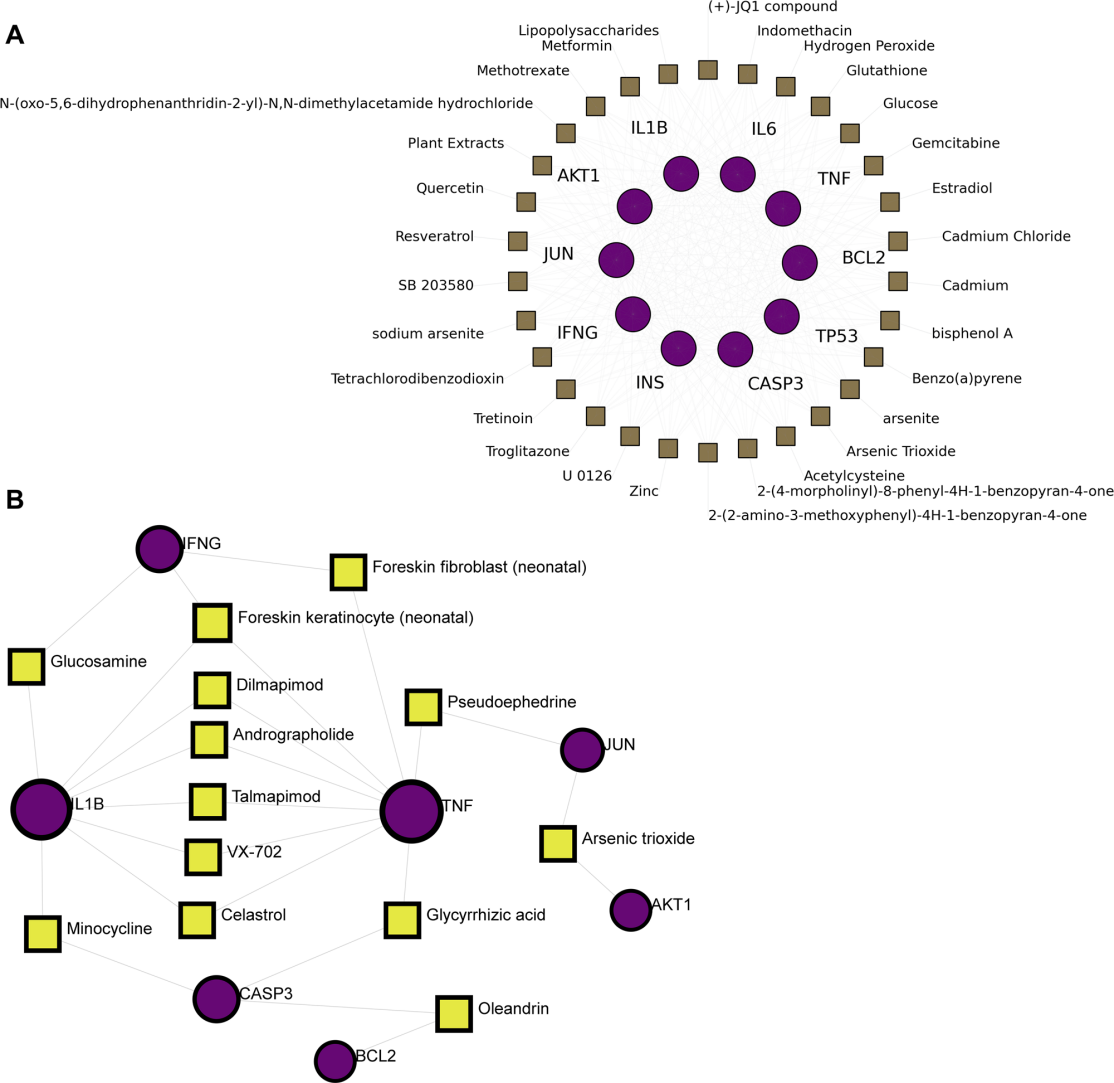
Integrative analysis of protein–chemical and protein–drug interaction networks. **A** Protein–chemical interaction network. Visualization of the interplay between the ten core proteins (circles) and chemical compounds (squares) retrieved from the Comparative Toxicogenomics Database (CTD). This network identifies key nodes, such as CASP3 and TNF, that integrate responses to environmental stressors and bioactive compounds, highlighting the susceptibility of the PD–UC axis to exogenous modulation. **B** Protein–drug interaction network. Mapping of therapeutic agents (squares) targeting the core proteins (circles) based on the DrugBank database. The network identifies several candidate drug-repurposing compounds, including anti-inflammatory modulators and kinase inhibitors, which may be evaluated for their potential to modulate pathways related to neurodegeneration and intestinal inflammation. All nodes were prioritized using an adaptive topological threshold (Degree >9 for chemicals; Degree >1 for drugs) to ensure the identification of functionally relevant therapeutic hubs. The network-based associations should be interpreted as prioritization of candidate compounds for future experimental evaluation rather than as direct evidence of therapeutic efficacy in PD–UC comorbidity.

The protein–chemical interaction network (Fig. 7A and Supplementary Data 5) linked the 10 core proteins to both environmental and endogenous chemicals. CASP3, TP53, BCL2, TNF, and IL6 emerged as high-connectivity hubs within this network. These hubs were connected to toxicants such as arsenic trioxide and acrolein, as well as to bioactive compounds including curcumin, highlighting potential interfaces between the shared PD–UC core module and chemical exposures. To further assess therapeutic tractability, we constructed a complementary protein–drug interaction network, in which TNF and IL1B showed the greatest connectivity to annotated drug associations (Fig. 7B and Supplementary Data 5). Using the same topological criteria, we prioritized several compounds with potential multi-target relevance, including minocycline, glucosamine, and apremilast, which were linked to key mediators such as TNF and IFNG. Overall, these results provide a network-based framework for prioritizing candidate compounds for future experimental repositioning studies.

## Discussion

By integrating transcriptomic datasets, disease-gene resources, and systems-level network analyses, this study identified a shared molecular landscape linking Parkinson’s disease (PD) and ulcerative colitis (UC). We identified 320 shared signature genes and a consensus-ranked 10-gene topology-derived module, together supporting convergent inflammatory and apoptotic regulatory programs potentially relevant to both neurodegeneration and intestinal inflammation. These findings provide computational support for a biologically plausible PD–gut connection that is consistent with, but does not prove, gut–brain axis models such as the gut-first hypothesis of PD^11–14^. Rather than indicating a single dominant pathway, the enrichment results point to a broader inflammatory-stress architecture characterized by microbial-response programs, lipopolysaccharide response, oxidative stress, and lipid/inflammatory signaling. Within this broader context, IL-17 signaling emerged as one biologically plausible pathway-level signal in the shared-signature analysis. Independent cohort-level testing, however, provided stronger support for this pathway in UC than in PD, whereas the AD comparator cohorts did not show reproducible significant enrichment. These comparator findings are therefore best interpreted as exploratory cross-disease context rather than definitive evidence of PD–UC specificity. This framework also helps explain the apparent mismatch between pathway-level and gene-level findings: IL-17 enrichment reflects coordinated perturbation across multiple pathway components, whereas single-gene AUC reflects the standalone discriminatory strength of individual genes, which may remain limited even when the pathway as a whole is perturbed.

Within this shared molecular landscape, several inflammatory and apoptosis-related genes occupied central topological positions, including *TNF, IL1B, IL6, AKT1, BCL2*, and *CASP3*. Collectively, these genes are consistent with a potential interface between chronic peripheral inflammation, epithelial injury, and neurodegenerative stress. Additional repeated cross-validated analyses in the discovery cohorts, followed by external validation, showed that the topology-derived core genes did not perform uniformly across datasets. *TNF, CASP3*, and *BCL2* displayed the most reproducible overall performance within the current evaluation framework and therefore emerged as the relatively stable components of the shared module. By contrast, *TP53, AKT1, IFNG, JUN*, and *INS* showed more context-dependent behavior, with moderate performance in one disease context but weaker generalizability in the other. Although IL1B and IL6 retained measurable signal in the PD framework, their performance was less consistent across the UC analyses, suggesting that they are better interpreted as contributors to pathway-level inflammatory activity than as uniformly reproducible standalone markers. Taken together, these findings indicate that network centrality should be interpreted primarily as evidence of regulatory relevance within the shared PD–UC molecular architecture rather than as uniform predictive utility. Accordingly, the 10-gene set is best viewed as a topology-derived shared regulatory module rather than as a finalized biomarker panel for clinical stratification. In this framework, these genes remain biologically informative even when their individual discriminatory performance varies across independent cohorts.

This interpretation is also consistent with the immune deconvolution analyses, which are compatible with a systems-level inflammatory connection between the two disorders rather than a narrowly diagnostic gene signature. The characterization of the peripheral immune landscape using blood-derived transcriptomic data suggests a systemic shift toward pro-inflammatory states in both disorders. In PD, the observed expansion of activated NK cells and their correlation with *TNF* and *IL1B* suggest that innate lymphoid cells may facilitate early systemic inflammation^15^. Conversely, the prevalence of neutrophils and monocytes in UC aligns with the signature of persistent systemic inflammatory stress^16^. Notably, reduced memory B-cell abundance observed across both diseases, together with inverse correlations between monocytes and T cell subsets, points to a conserved pattern of immune exhaustion. These findings are compatible with the possibility that PD and UC share elements of a dysregulated peripheral immune landscape.

Our multi-level regulatory analysis identified *TP53* and *JUN* as the primary transcriptional hubs within the shared genomic landscape^17,18^. The high degree and betweenness centrality of these TFs suggest that stress-activated and apoptosis-related programs may represent prominent regulatory influences within the shared network. Specifically, the central role of *JUN* aligns with its function in mediating neuronal apoptosis and mucosal inflammation, potentially contributing to stress-responsive transcriptional coordination across the gut–brain axis^19^. At the post-transcriptional level, the convergence of multiple microRNAs (miRNAs) on targets such as *BCL2* and *CASP3* indicates layered regulation of cell-fate pathways^20,21^. Among these, hsa-miR-21-5p displayed broad predicted regulatory coverage. miR-21 has been implicated in modulating intestinal epithelial barrier function and microglial activation states, positioning it as a potential mediator linking peripheral immune tone and neural inflammatory responses^22^. While causality cannot be inferred from network topology alone, this integrated regulatory architecture is consistent with a sustained inflammatory-stress regulatory state within the shared PD–UC molecular landscape.

Integration of protein–chemical and protein–drug interaction networks offers a systems-level perspective on the intersection between environmental exposures and the shared molecular architecture of PD and UC. Within the protein–chemical network, high-centrality nodes including CASP3, TP53, TNF, and IL6 occupied structurally influential positions, suggesting that inflammatory mediators may represent important interfaces through which exogenous or bioactive compounds intersect with the shared PD–UC network. Associations with toxicants such as arsenic trioxide and acrolein are biologically consistent with prior evidence linking these compounds to mitochondrial dysfunction and redox imbalance^23–25^. Conversely, bioactive compounds including curcumin and quercetin were mapped to high-degree regulatory nodes, providing a preliminary network-based rationale for their further investigation as potential modulators of inflammatory-stress pathways^26–29^. Pharmacological profiling likewise identified TNF and IL1B as prominent drug-interaction hubs, consistent with the broader inflammatory emphasis of the shared module and with prior epidemiological observations regarding anti-TNF therapy in IBD^30,31^. Among repositionable small molecules, minocycline and apremilast emerged as prioritized multi-target candidates^32,33^. Minocycline remains of interest because of its BBB permeability^34,35^, although prior clinical studies have yielded heterogeneous outcomes, underscoring the gap between mechanistic plausibility and clinical efficacy^32,36,37^. Overall, these findings should be interpreted as hypothesis-generating prioritization from an in silico network framework rather than as direct evidence for therapeutic benefit in PD–UC comorbidity.

Several limitations should be considered when interpreting these results. First, the current findings are primarily based on the integrated analysis of public transcriptomic datasets, and the identified signatures therefore remain computational in nature until validated experimentally. Although we incorporated external validation cohorts and additional cross-validated analyses to improve stability, direct wet-lab confirmation is still required. Second, several features of the cohort design constrain the generalizability of the findings. The PD discovery cohort (GSE22491) and PD validation cohort (GSE75249) both originated from France, albeit from distinct clinical and genetic backgrounds, which may limit the broader geographic generalizability of the PD-related signals. Third, the UC discovery cohort (GSE3365) was derived from PBMCs, whereas the UC validation cohort (GSE119600) used whole blood. This design offers a partial test of cross-sample consistency, but it also introduces potential confounding due to differences in leukocyte composition, which may influence the observed gene-expression patterns independently of disease biology. Fourth, although we expanded the AD comparator framework by including two blood-based Alzheimer’s disease cohorts (GSE97760 and GSE63060), these datasets were used only for the comparator context rather than as formal validation cohorts. Moreover, the IL-17 results across the AD comparators were non-significant and directionally inconsistent, and therefore should not be interpreted as definitive evidence regarding disease specificity. In addition, because one AD dataset (GSE63060) showed smaller transcriptomic effect sizes, a moderately relaxed fold-change threshold was applied to obtain sufficient genes for enrichment analysis, which may introduce additional variability when comparing across datasets. Fifth, the topology-derived core gene set represents network centrality within the shared PD–UC interactome rather than uniformly strong gene-level discriminatory performance. Cross-validated analyses demonstrated heterogeneous performance across genes, with some showing relatively stable signals and others exhibiting context-dependent or limited standalone reproducibility. This distinction should be considered when interpreting the core gene set as a shared regulatory module rather than a finalized diagnostic panel. Sixth, because our analyses relied on peripheral blood-derived transcriptomic data, they may not fully capture the spatial and temporal heterogeneity of the gut–brain axis, potentially overlooking tissue-specific dynamics within the colonic mucosa or the substantia nigra^38^. Future studies utilizing single-cell RNA sequencing or spatial transcriptomics may help resolve the cellular context of these shared signals^39^. Finally, although our results indicate a potential molecular correlation between PD and UC, transcriptomic associations do not inherently establish direct causality. Current Mendelian randomization studies have yielded inconsistent genetic links, suggesting that environmental factors, epigenetic modifications, or microbiome–host interactions may play a more dominant role in their comorbidity^40–42^. Ultimately, translating these hub genes into clinically actionable targets will require rigorous validation across diverse populations and the development of preclinical models that accurately recapitulate the complex PD–UC interface.

## Methods

**Fig. 8:**
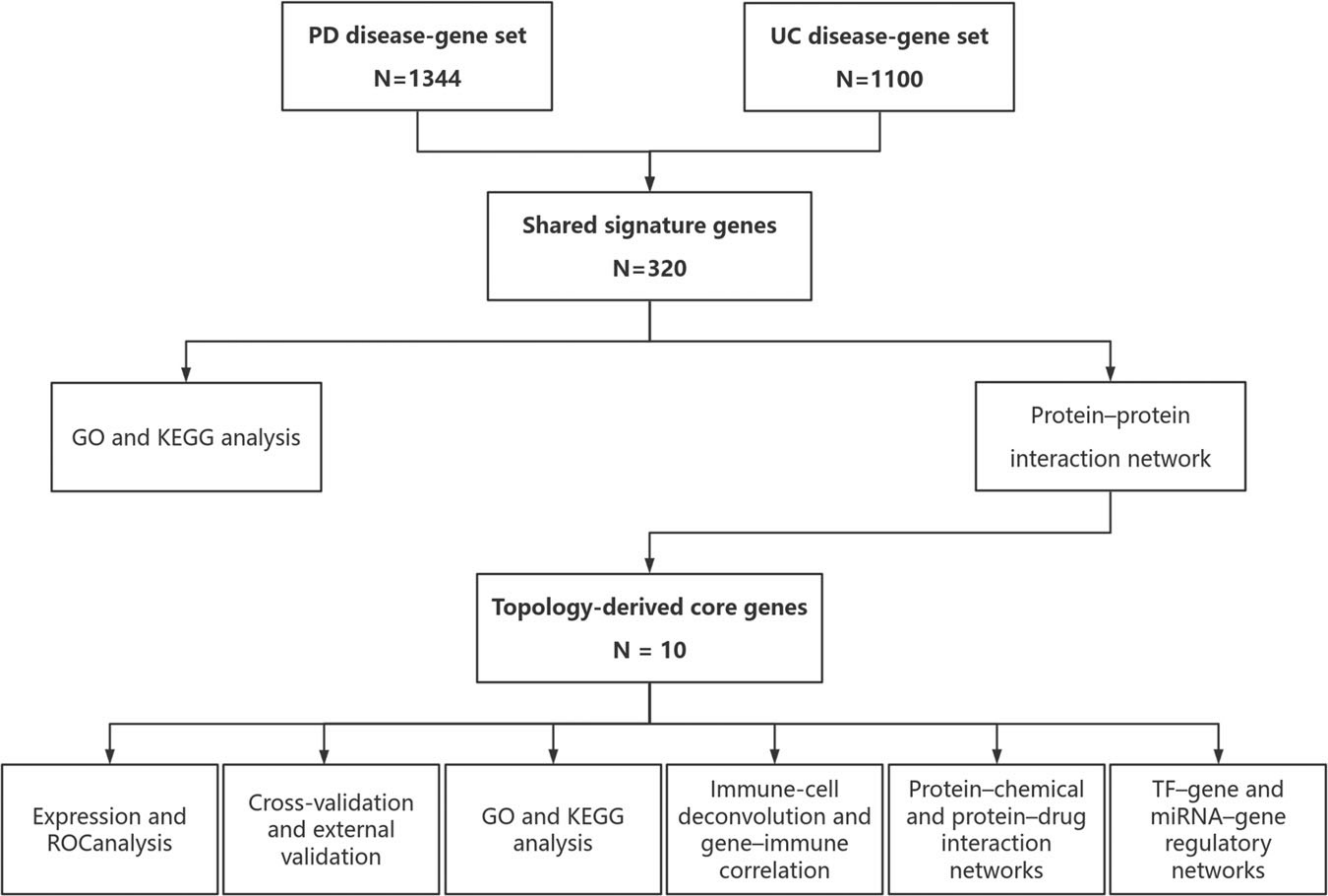
Integrated bioinformatics workflow for identifying shared gene signatures between Parkinson’s disease and ulcerative colitis. The flowchart illustrates the sequential steps of the study, including multi-omics data integration from databases, identification of shared signature genes, protein-protein interaction (PPI) network construction, and subsequent enrichment and validation analyses.

This study was reported in accordance with the Strengthening the Reporting of Genetic Association Studies (STREGA) recommendations, an extension of the STROBE Statement. The completed STREGA checklist is provided in Supplementary Table 3. An overview of the integrated bioinformatics workflow used in this study is shown in Fig. 8.

### Data acquisition

To investigate the molecular intersections between PD and UC, we integrated genomic evidence from disease-specific repositories with transcriptomic profiles from public archives. Genomic associations were retrieved on February 10, 2026, from DisGeNET (https://www.disgenet.org, v7.0)^43^, the Comparative Toxicogenomics Database (CTD, https://ctdbase.org/)^44^, and GeneCards (https://www.genecards.org/, v5.26)^45^. To prioritize high-confidence candidates and ensure the selection of biologically established markers, we extracted the top 500 genes from each repository based on their respective relevance or inference scores for PD and UC, thereby establishing a genomic reference set for downstream intersection analysis.

Transcriptomic datasets were curated from the Gene Expression Omnibus (GEO, https://www.ncbi.nlm.nih.gov/geo/)^46^ according to a multi-stage selection framework designed to ensure high data quality and biological comparability. We restricted inclusion to peripheral immune-cell samples, specifically peripheral blood mononuclear cells (PBMCs) or whole blood, to align with the systemic inflammatory characteristics of the PD–UC axis and minimize spatial heterogeneity associated with localized brain or colonic tissue biopsies. Selection criteria further required the presence of clearly defined clinical diagnostic standards, the availability of standardized raw or processed data for reanalysis, and the use of widely validated expression platforms—including Agilent, Affymetrix, and Illumina—to mitigate technical noise during cross-platform integration.

Following these criteria, six independent cohorts from four countries (France, the USA, Poland, and the United Kingdom) were included, as summarized in Supplementary Table 1. For clarity, GSE22491 (PD) and GSE3365 (UC) were used as discovery cohorts, GSE75249 (PD) and GSE119600 (UC) as external validation cohorts, and GSE97760 together with GSE63060 as biological comparator cohorts rather than formal validation datasets. The PD discovery cohort, GSE22491 (France, Agilent GPL6480) included 10 patients with PD and 8 healthy controls (HC)^47^, whereas the PD validation cohort, GSE75249 (France, Agilent GPL4133), included 14 PD and 13 HC^48^. Although both PD cohorts originated from France, they represented distinct clinical and genetic backgrounds, with GSE22491 including LRRK2-associated PD cases and GSE75249 including ATXN2 expansion carriers or sporadic PD cases. The UC discovery cohort, GSE3365 (USA, Affymetrix GPL96)^49^, comprised 26 UC and 42 HC PBMC samples, whereas the UC validation cohort, GSE119600 (Poland, Illumina GPL10558), comprised 93 UC and 47 HC whole-blood samples^50^. This PBMC-to-whole-blood design allowed a limited assessment of cross-sample consistency, while also necessitating caution because differences in leukocyte composition may influence observed gene-expression patterns. Finally, two AD cohorts were included to provide preliminary context for pathway-level specificity rather than as formal validation cohorts. GSE97760 (USA, Agilent GPL16699)^51^, included 9 patients with AD and 10 HC whole-blood samples. To further strengthen this biological comparison, we added GSE63060 (UK, Illumina GPL6947)^52^, a large-scale dataset from the AddNeuroMed Cohort consisting of 145 AD patients and 104 age- and sex-matched controls.

### Differential expression analysis and signature identification

DEGs expression analysis was performed using the GEO2R interface (https://www.ncbi.nlm.nih.gov/geo/geo2r/), which implements the *limma* R package (v3.58.1). Expression data were log2-transformed and quantile-normalized as appropriate within the GEO2R/limma workflow to improve comparability and stabilize variance. When multiple probes mapped to the same gene symbol, probes were collapsed by retaining the probe with the highest mean expression value. DEGs were identified using the empirical Bayes moderation method, with a stringent threshold of FDR < 0.00001 and |log₂ Fold-Change| > 1.

To define disease-specific signature genes, we used a multistep integration strategy. For both PD and UC, we first generated disease-specific gene catalogs by taking the union of DEGs identified in the discovery cohorts and the top 500 genes retrieved from the genomic repositories described above (DisGeNET, CTD, and GeneCards). This approach ensured that each disease signature incorporated both transcriptomic evidence and curated disease-associated genes. Shared signature genes were then identified by intersecting the refined PD and UC catalogs, with the overlap visualized using a Venn diagram^53^. His shared gene set served as the basis for all downstream network construction and functional enrichment analyses.

For the AD comparator analyses, differential-expression thresholds were used for exploratory comparison only and were not used to define the PD–UC shared signature genes. GSE97760 was analyzed using the same threshold applied in the PD and UC discovery cohorts (FDR < 0.00001 and |log₂FC| > 1). For GSE63060, however, the fold-change cutoff was relaxed to |log₂FC| > 0.58 because the blood-derived AD transcriptomic signal showed substantially smaller effect sizes, and the stricter cutoff yielded too few genes for informative downstream enrichment analysis. This modification was restricted to the comparator analysis and did not affect the derivation of the 320 shared PD–UC signature genes.

### Network topology and core gene screening

To characterize the shared PPI landscape between PD and UC, we constructed a PPI network from the shared signature genes using STRING (v12.0) with a medium-confidence score cutoff of 0.4^54^. To reduce the influence of ubiquitous, high-abundance proteins that might dominate network topology without being specific to disease biology, we excluded housekeeping-associated markers, namely *GAPDH, ACTB*, and *ALB*, before topological analysis. The resulting network was visualized and analyzed in Cytoscape (v3.10.4)^55^.

To identify topology-derived hub genes within the shared interactome, we used a consensus-ranking strategy implemented in the CytoHubba plugin (v0.1)^56^. Five complementary centrality algorithms were evaluated: Maximal Clique Centrality (MCC), Stress, Maximum Neighborhood Component (MNC), Degree, and Betweenness Centrality. Genes that ranked within the top 20 across all five metrics were retained as candidate hubs, thereby prioritizing nodes with both strong local connectivity and broader topological influence within the network.

This multi-algorithm intersection yielded 10 consensus core genes. These genes were defined primarily by shared network centrality within the PD–UC interactome rather than by uniformly strong discriminatory performance across all datasets. To further explore their functional relationships, we used the GeneMANIA plugin (https://genemania.org/, v 3.5.3)^57^ to generate an evidence-based interaction network integrating co-expression, physical interactions, and predicted functional links.

To assess whether the topology-derived core genes also showed reproducible discriminatory performance, we conducted repeated stratified 5-fold cross-validation separately in the PD and UC discovery cohorts. For each core gene, a univariate logistic regression model was fitted within the training folds, and mean cross-validated AUC values with empirical 95% intervals were calculated across repeated iterations. External validation was then performed in the corresponding PD and UC validation cohorts using the same single-gene framework. This analysis was intended to evaluate predictive stability rather than to develop a finalized diagnostic classifier.

### Functional enrichment and comparator analysis

To characterize the biological functions of the shared signature genes and topology-derived core genes, we performed Gene Ontology (GO) and Kyoto Encyclopedia of Genes and Genomes (KEGG) enrichment analyses using the online platform (https://www.bioinformatics.com.cn)^58^. GO terms were evaluated across the Biological Process (BP), Cellular Component (CC), and Molecular Function (MF) categories to provide a multidimensional functional profile, and KEGG pathway annotations were analyzed using the same platform. Statistical significance was defined as a Benjamini-Hochberg-adjusted *P* < 0.05.

Because the AD datasets served as biological comparator cohorts rather than as components of the PD–UC shared-signature derivation, enrichment results involving AD were analyzed separately and interpreted comparatively. For these exploratory comparator analyses, DEG sets from each AD cohort were subjected to GO/KEGG enrichment analysis to assess whether the broader inflammatory and lipid-associated patterns observed in the shared PD–UC signature were also reproducibly present in AD blood transcriptomes.

To further assess whether the IL-17 pathway signal identified in the discovery analysis could be independently reproduced, we performed cohort-level pathway enrichment analysis using a fixed KEGG IL-17 gene set (hsa04657) across the PD and UC discovery cohorts, their corresponding validation cohorts, and two AD blood-based comparator cohorts (GSE97760 and GSE63060). This analysis was intended to evaluate pathway-level reproducibility and comparator context rather than to establish formal disease specificity. Because the comparator analyses were exploratory and one AD cohort (GSE63060) required a moderately relaxed fold-change threshold to yield sufficient genes for downstream enrichment analysis, the AD results were interpreted cautiously and were not treated as threshold-equivalent counterparts of the PD–UC discovery framework.

### Immune cell composition estimation

We estimated the relative proportions of 22 leukocyte subsets using the LM22 signature matrix in the CIBERSORTx cloud platform (https://cibersortx.stanford.edu/)^59^. Gene expression matrices were preprocessed to ensure unique gene mapping and data integrity before deconvolution. To balance computational efficiency with statistical power, 100 permutations were used to estimate deconvolution significance and generate sample-level *P* values. Differences in cell fractions between patients and healthy controls were evaluated using the Wilcoxon rank-sum test. Associations between the 10 core genes and estimated immune-cell proportions were assessed using Spearman’s rank correlation. *P* values were adjusted for multiple comparisons using the Benjamini-Hochberg method, and adjusted *P* < 0.05 was considered statistically significant. Statistical analyses and data visualization not otherwise specified were performed in R (v4.4.1) using ggplot2 (v4.0.2), ggpubr (v0.6.2), and pheatmap (v1.0.13).

### Regulatory and pharmacological network construction

Transcriptional and post-transcriptional regulatory networks were constructed using NetworkAnalyst 3.0 platform (https://www.networkanalyst.ca/)^60^. Upstream TFs and miRNAs regulating the 10 core genes were predicted from the OmniPath^61^ and RegNetwork databases^62^. To prioritize influential regulators, we applied degree-based filtering thresholds (degree > 9 for TFs, degree > 3 for miRNAs, and degree > 3 for integrated networks) together with a betweenness centrality threshold > 0.

Pharmacological networks were constructed using protein-chemical interaction data from CTD^44^ and protein-drug interaction data from DrugBank 5.0^63^. To account for structural differences between network types, we applied an adaptive thresholding strategy. Hub nodes were defined using degree > 9 for chemicals or degree > 1 for drugs, together with betweenness centrality > 0. This approach prioritized nodes that could serve as bridges for information flow within the shared PD-UC network.

### Ethics approval and consent to participate

All data used in this study were obtained from publicly available databases. The GEO datasets included in this analysis (GSE22491, GSE3365, GSE75249, GSE119600, GSE63060, and GSE97760) were originally collected with approval from their respective institutional review boards and with informed consent from all participants. Use of these de-identified, publicly accessible data was consistent with GEO access policies and did not require additional ethical approval or participant consent. The other databases used in this study, including DisGeNET, CTD, GeneCards, STRING, and DrugBank, were publicly accessible resources that aggregate anonymized data from previously published studies and did not involve direct interaction with human subjects.

## Supplementary information

supplementary-revised

## Data Availability

The datasets analyzed in this study are publicly available in the GEO repository. The GEO accession numbers used in this study are GSE22491, GSE3365, GSE75249, GSE119600, GSE97760, and GSE63060. All analyzed data were derived from publicly accessible, de-identified bioinformatics resources. The supplementary data supporting the findings of this study (Supplementary Data 1–5) have been deposited in Figshare under the 10.6084/m9.figshare.31423433. Custom code used for data processing and analysis is available from the corresponding author upon reasonable request.
