# Supplementary material for: Shared gene signatures and biochemical regulatory networks linking Parkinson’s disease and ulcerative colitis
Source: NPJ Parkinsons Dis. 2026 May 6;12:109. doi: 10.1038/s41531-026-01374-z (PMC13149972; doi:10.1038/s41531-026-01374-z)
Supplement: Supplementary file 1 — supplementary-revised [file 41531_2026_1374_MOESM1_ESM.pdf]

Shared Gene Signatures and Biochemical Regulatory Networks Linking Parkinson's Disease and Ulcerative Colitis

Xiaohui Sun[1], Zhichen An[2], Shufei Wang[3], Kongjia Wang[4]

[1] Department of Neurology, Beijing Tsinghua Changgung Hospital, School of Clinical Medicine, Tsinghua University, Beijing, China

[2] Orthopedic Sports Medicine Center, Beijing Tsinghua Changgung Hospital, School of Clinical Medicine, Affiliated Hospital of Tsinghua University, Beijing, China.

[3] College of Clinical and Basic Medical Sciences, Shandong First Medical University & Shandong Academy of Medical Sciences, Jinan, China

[4] Department of Clinical Sciences, Lund University, Clinical Research Centre (CRC), Box 50332, SE-20213 Malmö, Sweden

* Corresponding authors:

Kongjia Wang 0009-0002-0995-409X

kongjia.wang@med.lu.se

Department of Clinical Sciences, Lund University, Clinical Research Centre (CRC), Box 50332, SE-20213 Malmö, Sweden

Keywords: Parkinson's disease, ulcerative colitis, Gut-brain Axis, bioinformatics analysis, immunology

Content

Shared Gene Signatures and Biochemical Regulatory Networks Linking Parkinson's Disease and Ulcerative Colitis 1

Content 2

1 Supplementary Tables 4

Supplementary Table 1. Summary of the GEO datasets included in this study. 4

Supplementary Table 2. Topological ranking of candidate core genes across multiple cytoHubba algorithms. 4

Supplementary Table 3. STREGA Reporting Recommendations (Extended from STROBE Statement). 5

Supplementary Table 4. Independent cohort-level GSEA summary for the KEGG IL-17 signaling pathway across discovery, validation, and comparator datasets 14

Supplementary Table 5. Top-ranked enrichment terms in AD comparator cohorts 15

2 Supplementary Figures 16

Supplementary Figure 1. Descriptive expression and external validation profiles of topology-derived core genes in PD and UC cohorts. 17

Supplementary Figure 2. Independent immune-cell validation in PD and UC cohorts. 20

Supplementary Figure 3. Sensitivity analysis and functional characterization of shared Parkinson's disease and ulcerative colitis signatures based on relaxed statistical thresholds. 21

Supplementary Figure 4. Cross-validated and external validation assessment of topology-derived core genes in Parkinson's disease and ulcerative colitis cohorts. 22

Supplementary Figure 5. Independent cohort-level evaluation of IL-17 signaling pathway enrichment across discovery, validation, and comparator datasets 23

Supplementary Figure 6. Transcriptional and post-transcriptional regulatory architecture of PD–UC core genes. 25

Supplementary Figure 7. Integrative analysis of protein–chemical and protein–drug interaction networks. 26

3 Supplementary Data 27

Supplementary Data 1. Differential Expression Gene Lists 27

Supplementary Data 2. Shared Signature Genes (320 Genes) and Evidence Sources 27

Supplementary Data 3. Functional Enrichment Results (GO/KEGG) 27

Supplementary Data 4. Immune Infiltration Profiles (CIBERSORTx) 28

Supplementary Data 5. Regulatory Networks and Therapeutic Candidates 28

1 Supplementary Tables

Supplementary Table 1. Summary of the GEO datasets included in this study.

Dataset (GSE ID)	Role	Disease	Region	Platform	Sample Source	Sample Size (Dis/Con)	PMID
**GSE22491**	Discovery	PD	France	Agilent GPL6480	PBMC	10 PD / 8 HC	20096956
**GSE3365**	Discovery	UC	USA	Affymetrix GPL96	PBMC	26 UC / 42 HC	16436634
**GSE75249**	Validation	PD	France	Agilent GPL4133	PBMC	14 PD / 13 HC	27663142
**GSE119600**	Validation	UC	Poland	Illumina GPL10558	Whole Blood	93 UC / 47 HC	31076612
**GSE97760**	Comparator	AD	USA	Agilent GPL16699	Whole Blood	9 AD / 10 HC	25079797
**GSE63060**	Comparator	AD	UK	Illumina GPL6947	Whole Blood	145 AD / 104 HC	26343147

GSE22491: PD patients carrying the LRRK2 G2019S mutation.
GSE75249: PD patients with/without ATXN2 expansions, characterized by RNA-binding disturbances.

Supplementary Table 2. Topological ranking of candidate core genes across multiple cytoHubba algorithms.

Rank	MCC	Betweenness	Stress	MNC	Degree
1	*IL6*	*AKT1*	*TNF*	*TNF*	*TNF*
2	*AKT1*	*TP53*	*IL6*	*AKT1*	*AKT1*
3	*IL1A*	*TNF*	*TP53*	*IL6*	*IL6*
4	*CCL2*	*IL6*	*AKT1*	*TP53*	*TP53*
5	*BCL2*	*INS*	*IL1B*	*IL1B*	*IL1B*
6	*IL1B*	*IL1B*	*INS*	*INS*	*INS*
7	*CASP3*	*EGFR*	*IL2*	*CASP3*	*CASP3*
8	*PTGS2*	*IL2*	*EGFR*	*BCL2*	*BCL2*
9	*NFKB1*	*MYC*	*CYCS*	*JUN*	*JUN*
10	*STAT3*	*MAPK3*	*JUN*	*EGFR*	*EGFR*
11	*MMP9*	*JUN*	*MYC*	*STAT3*	*STAT3*
12	*IFNG*	*ESR1*	*MAPK3*	*HIF1A*	*HIF1A*
13	*HIF1A*	*FOS*	*FOS*	*MAPK3*	*MAPK3*
14	*TLR4*	*IFNG*	*CASP3*	*IFNG*	*IFNG*
15	*INS*	*CASP3*	*ESR1*	*NFKB1*	*NFKB1*
16	*TGFB1*	*SOD1*	*IFNG*	*MYC*	*MYC*
17	*CXCL8*	*CD4*	*SOD1*	*CTNNB1*	*CTNNB1*
18	*JUN*	*BCL2*	*CD4*	*PTGS2*	*PTGS2*
19	*TNF*	*CYCS*	*BCL2*	*MMP9*	*MMP9*
20	*TP53*	*HIF1A*	*PRKCA*	*TLR4*	*TLR4*

Note: Candidate genes were ranked using the cytoHubba plugin in Cytoscape 3.10.1. The top 20 genes from five representative algorithms (MCC, Betweenness, Stress, MNC, and Degree) are displayed. The final

ten core hub genes were identified based on their consistent high ranking (top 20) across at least four topological methods, ensuring the robustness of the biological signatures against algorithmic bias.

Supplementary Table 3. STREGA Reporting Recommendations (Extended from STROBE Statement).

Item No.	STROBE Guideline	Extension for Genetic Association Studies (STREGA)	Location in Manuscript (Page)	Response / Details
**Title and Abstract**				
1	(a) Indicate the study's design with a commonly used term in the title or abstract.	(a) State if the study is the first report of a genetic association or a replication effort, or both.	Title (p. 1); Abstract (p. 2)	Exploratory integrative bioinformatics study using public disease-gene and transcriptomic resources; discovery-oriented rather than replication.
	(b) Provide in the abstract an informative and balanced summary of what was done and what was found.	(b) Indicate in the abstract whether the study reports results from a **pre-planned** or **post-hoc** analysis, and whether data were collected **specifically for this study** or obtained from **existing databases**.	Abstract (p. 2)	Post-hoc analysis of existing public datasets (DisGeNET, CTD, GeneCards, GEO); no pre-specified genetic hypothesis beyond exploring shared PD–UC signatures.
**Introduction**				
2	Background/rationale: Explain the scientific background and rationale for the investigation being reported.	(a) State the **gene, variant, or haplotype under investigation** and its **putative functional role**. (b) State whether the study is	Introduction (p. 2-3)	Gut–brain axis and PD–UC comorbidity motivate the study; not a candidate-gene, fine-mapping, or GWAS study, but an integrative bioinformatics analysis.

Item No.	STROBE Guideline	Extension for Genetic Association Studies (STREGA)	Location in Manuscript (Page)	Response / Details
		a **candidate gene study**, a **fine-mapping study**, or a **genome-wide association study (GWAS)**.		
3	Objectives: State specific objectives, including any pre-specified hypotheses.	State specific **genetic associations** to be examined, and whether they are **primary or secondary** objectives.	Introduction (p. 3)	Objective: identify shared signature genes, topology-derived core genes, immune/regulatory features, and pathway-level links between PD and UC; exploratory aim.
**Methods**				
4	Study design: Present key elements of study design early in the paper.	(a) Describe the **study design** (e.g., case-control, cohort, cross-sectional, family-based). (b) If a **genetic association study**, describe the **sampling strategy** (e.g., population-based, hospital-based).	Methods (p. 14)	Case-control integrative bioinformatics study using existing public blood-derived transcriptomic cohorts with healthy controls.
5	Setting: Describe the setting, locations, and relevant dates, including periods of recruitment, exposure, follow-up, and data collection.	(a) Describe the **geographic location** and **ethnicity** of participants, if available. (b) Describe **dates of recruitment** and **data collection**.	Methods (p. 14-16)	Cohorts from France, USA, Poland, and UK; PBMC or whole blood; ethnicity not uniformly reported in source datasets.

Item No.	STROBE Guideline	Extension for Genetic Association Studies (STREGA)	Location in Manuscript (Page)	Response / Details
6	Participants: (a) Give the eligibility criteria, and the sources and methods of selection of participants. Describe methods of follow-up. (b) For matched studies, give matching criteria and number of exposed and unexposed.	(a) Specify **inclusion and exclusion criteria** for cases and controls. (b) Report **numbers of cases and controls** and whether they were **matched** on any variables.	Methods (p. 15-16)	Inclusion: public datasets with clear diagnosis, blood-derived samples, analyzable expression data, standard platforms; datasets used as provided; matching not controlled by current study.
7	Variables: Clearly define all outcomes, exposures, predictors, potential confounders, and effect modifiers. Give diagnostic criteria, if applicable.	(a) Define the **genetic exposure** (gene, variant, haplotype) and how it was measured (genotyping method, quality control). (b) Define **outcome** (d isease) with diagnostic criteria.	Methods (p. 14-16)	No direct genotyping; exposure operationalized through curated disease-gene resources and transcriptomic signals; outcomes were PD, UC, or AD comparator status.
8	Data sources/measurement: For each variable of interest, give sources of data and details of methods of assessment (measurement). Describe comparability of assessment	(a) Describe **genotyping platform**, **laboratory methods**, **quality control** procedures. (b) Report whether genotyping was performed in **batches**, and if cases and controls	Methods (p. 14-20)	GEO transcriptomic platforms (Agilent, Affymetrix, Illumina); differential expression by limma; enrichment by GO/KEGG/GSEA; immune deconvolution by CIBERSORTx; no direct genotyping.

Item No.	STROBE Guideline	Extension for Genetic Association Studies (STREGA)	Location in Manuscript (Page)	Response / Details
	methods if there is more than one group.	were interspersed.		
9	Bias: Describe any efforts to address potential sources of bias.	(a) Describe **populatio n stratification** assessment and correction methods, if applicable. (b) Report **Hardy-Weinberg equilibrium** tests for variants.	Methods (p. 17-19); Discussion (p. 12-14)	Population stratification not assessed due to absence of genotype data; bias addressed by discovery/validation/comparator design, exclusion of housekeeping hubs, multiple-testing correction, and cross-validation.
10	Study size: Explain how the study size was arrived at.	(a) Report the **number of participants** and **number of genotyped variants** after quality control.	Methods (p. 15-16)	Sample size determined by eligible public datasets; no genotyped variants were generated in this study.
11	Quantitative variables: Explain how quantitative variables were handled in the analyses. If applicable, describe which groupings were chosen and why.	(a) For **quantitative traits**, describe how they were analyzed (e.g., as continuous or categorical). (b) If **allele frequencies** or **genotype counts** are reported, state how they were estimated.	Methods (p. 16-19)	Gene expression analyzed as continuous log2-transformed values; AUC, NES, immune fractions, and correlations treated as continuous measures.
12	Statistical methods: (a) Describe all statistical methods,	(a) State whether **one-sided** or **two-sided** p-values are reported, and	Methods (p. 16-20)	Two-sided tests; limma for DEGs; Wilcoxon for immune-cell differences; Spearman for correlations; repeated

Item No.	STROBE Guideline	Extension for Genetic Association Studies (STREGA)	Location in Manuscript (Page)	Response / Details
	including those used to control for confounding. (b) Describe any methods used to examine subgroups and interactions. (c) Explain how missing data were addressed. (d) If applicable, describe analytical methods taking account of sampling strategy. (e) Describe any sensitivity analyses.	whether adjustments for multiple comparisons were made. (b) Describe **methods for testing associations** (e.g., logistic regression, linear regression, transmission disequilibrium test). (c) If **haplotype analysis** was performed, describe methods. (d) If **gene-gene** or **gene-environment** inter actions were examined, describe methods. (e) If **multiple testing** was corrected, specify method (e.g., Bonferroni, FDR).		stratified 5-fold CV and external validation for core genes; BH/FDR correction applied.
**Results**				
13	Participants: (a) Report numbers of individuals at each stage of study—e.g., numbers potentially eligible, examined for eligibility, confirmed eligible, included in the study,	(a) Report **numbers of cases and controls** included in the final analysis, and reasons for exclusion. (b) Report **genotyping success rates** and **call rates** if applicable.	Methods (p. 15-16); Results (p. 3)	Discovery, validation, and comparator cohort sizes are reported; no additional exclusions beyond dataset eligibility.

Item No.	STROBE Guideline	Extension for Genetic Association Studies (STREGA)	Location in Manuscript (Page)	Response / Details
	completing follow-up, and analyzed. (b) Give reasons for non-participation at each stage. (c) Consider use of a flow diagram.			
14	Descriptive data: (a) Give characteristics of study participants (e.g., demographic, clinical, social) and information on exposures and potential confounders. (b) Indicate number of participants with missing data for each variable of interest.	(a) Report **demographic and clinical characteristics** of cases and controls (age, sex, ethnicity, disease duration, etc.). (b) Report **genotype distributions** and **allele frequencies** for cases and controls.	Methods (p. 15-16)	Available cohort descriptors include country, platform, sample source, and sample size; detailed subject-level demographics were not uniformly available in GEO.
15	Outcome data: Report numbers of outcome events or summary measures.	(a) Report **number of cases** and **controls** for each genotype group (if applicable).	Results (p. 3); Table 1 (p. 26)	Outcome groups were PD, UC, or AD comparator versus healthy controls; no genotype groups analyzed.
16	Main results: (a) Give unadjusted estimates and, if applicable, confounder-adjusted estimates and their precision	(a) Report **unadjusted and adjusted genetic effect estimates** (odds ratios, beta coefficients, etc.) with confidence	Results (p. 4-9); Tables 2-4 (p. 26-28)	Main outputs include log2FC, adjusted P values, discovery/validation AUCs, cross-validated and external validation AUCs with intervals/CIs, and

Item No.	STROBE Guideline	Extension for Genetic Association Studies (STREGA)	Location in Manuscript (Page)	Response / Details
	(e.g., 95% confidence intervals). Make clear which confounders were adjusted for and why they were included. (b) Report category boundaries when continuous variables were categorized. (c) If relevant, consider translating estimates of relative risk into absolute risk for a meaningful time period.	intervals. (b) Report **p-values** for associations, and whether they were **corrected for multiple testing**. (c) For **quantitative traits**, report the **effect size** per allele or genotype.		enrichment statistics.
17	Other analyses: Report other analyses done—e.g., analyses of subgroups and interactions, and sensitivity analyses.	(a) Report any **subgroup analyses** (e.g., by sex, age, disease subtype). (b) Report any **gene-gene** or **gene-environment** inter action analyses. (c) Report **haplotype analyses** if performed. (d) Report **sensitivity analyses** (e.g., excluding outliers, adjusting for population stratification).	Results (p. 5-9)	Additional analyses included cross-validation, external validation, IL-17 pathway testing in validation/comparator cohorts, immune deconvolution, gene–immune correlations, and regulatory/drug network analyses.
**Discussion**				

Item No.	STROBE Guideline	Extension for Genetic Association Studies (STREGA)	Location in Manuscript (Page)	Response / Details
18	Key results: Summarise key results with reference to study objectives.	(a) Summarise the **main genetic findings** in relation to the study objectives.	Discussion (p. 9-10)	Key findings: 320 shared signature genes, 10 topology-derived core genes, broader inflammatory-stress architecture, partial IL-17 support, immune changes, and regulatory/druggability signals.
19	Limitations: Discuss limitations of the study, taking into account sources of potential bias or imprecision. Discuss both direction and magnitude of any potential bias.	(a) Discuss **limitation s related to genotyping** (e.g., genotyping error, population stratification). (b) Discuss **limitation s related to phenotype definition** (e.g., disease heterogeneity). (c) Discuss **multiple testing burden** and how it was addressed.	Discussion (p. 12-14)	Limitations include public blood-derived datasets, no direct genotyping, French PD cohort concentration, PBMC vs whole-blood heterogeneity, comparator-only AD analyses, and limited disease-specificity inference.
20	Interpretation: Give a cautious overall interpretation considering objectives, limitations, multiplicity of analyses, results from similar studies, and other relevant evidence.	(a) Interpret the **genetic findings** in the context of current knowledge, and discuss whether they are **biologically plausible**. (b) Discuss whether the findings are **consistent with previous reports** or **novel**.	Discussion (p. 9-13)	Findings are interpreted as a shared inflammatory/apoptotic regulatory module linking PD and UC, not a finalized biomarker panel; IL-17 shows stronger support in UC than PD.

Item No.	STROBE Guideline	Extension for Genetic Association Studies (STREGA)	Location in Manuscript (Page)	Response / Details
21	Generalisability: Discuss the generalisability (external validity) of the study results.	(a) Discuss the **generalizability** of genetic associations to other populations, and whether **ethnic-specific effects** may exist.	Discussion (p. 13-14)	Generalizability is limited by geographic concentration of PD cohorts, sample-source heterogeneity, and incomplete ethnicity/covariate information; broader validation is needed.
**Other Information**				
22	Funding: Give the source of funding and the role of the funders for the present study and, if applicable, for the original study on which the present article is based.	(a) State the **sources of funding** and **role of funders** for the genetic study. (b) If the study used **pre-existing data**, state the funding sources for the original data collection.	Acknowledgements (p. 21)	No specific funding for the present study; source datasets were generated by original investigators.
(Additional STREGA items)				
**A1**	Population stratification	Describe assessment/control efforts	Methods (p. 17-19); Discussion (p. 12-14)	Not directly assessed because no genotype data were available; limitation explicitly acknowledged.
**A2**	Hardy-Weinberg equilibrium	Report HWE if applicable	Not applicable	No genotype or variant-level analysis was performed.

Item No.	STROBE Guideline	Extension for Genetic Association Studies (STREGA)	Location in Manuscript (Page)	Response / Details
**A3**	Multiple testing	Specify correction method and scope	Methods (p. 16-20)	Differential expression used stringent FDR threshold; enrichment, immune, and correlation analyses used Benjamini–Hochberg correction.
**A4**	Replication	If replication, describe original study/criteria	Not applicable	This is an exploratory discovery study with external validation and AD comparator analyses, not a formal replication study.
**A5**	Data access	Describe restrictions on data/material availability	Data Availability (p. 20-21)	All primary datasets are publicly available from GEO and cited bioinformatics resources; supplementary data are deposited in Figshare.

Notes:

- Items marked "Not applicable" indicate that the study did not involve genotyping or direct participant data.
- The study is an integrative bioinformatics analysis using pre-existing genomic and transcriptomic data, not a traditional genetic association study with individual genotyping. However, STREGA is applied to ensure transparent reporting of methods and findings relevant to genetic associations.

Supplementary Table 4. Independent cohort-level GSEA summary for the KEGG IL-17 signaling pathway across discovery, validation, and comparator datasets

Dataset	Disease	Role	NES	Nominal P value	FDR-adjusted P value	Interpretation
**GSE3365**	UC	Discovery	2.069	1.71×10^{-6}	1.71×10^{-6}	Significant enrichment
**GSE22491**	PD	Discovery	1.158	0.193	0.193	Positive but non-significant
**GSE119600**	UC	Validation	1.384	0.0255	0.0255	Significant enrichment
**GSE75249**	PD	Validation	1.040	0.400	0.400	Positive but non-significant
**GSE97760**	AD	Comparator	1.100	0.280	0.280	Positive but non-significant
**GSE63060**	AD	Comparator	-1.115	0.233	0.233	Negative but non-significant

Abbreviations:
AD, Alzheimer's disease; FDR, false discovery rate; GSEA, gene set enrichment analysis; NES, normalized enrichment score; PD, Parkinson's disease; UC, ulcerative colitis.

Table note:
Preranked gene set enrichment analysis (GSEA) was performed independently in the discovery, validation, and comparator cohorts using a fixed KEGG IL-17 signaling pathway gene set (hsa04657). Genes were ranked according to differential expression between disease and control samples. Positive normalized enrichment score (NES) values indicate relative enrichment of IL-17-related genes toward the upregulated end of the ranked gene list. Significant enrichment (FDR-adjusted P < 0.05) was observed in the UC discovery (GSE3365) and validation (GSE119600) cohorts, whereas the PD discovery (GSE22491), PD validation (GSE75249), and both AD comparator cohorts (GSE97760, GSE63060) were non-significant, with GSE63060 showing a negative NES. The AD comparator framework should be interpreted cautiously because it was exploratory, one comparator cohort was small, and the two AD datasets showed heterogeneous enrichment directions.

Supplementary Table 5. Top-ranked enrichment terms in AD comparator cohorts

Dataset	Category	Term ID	Description	Count	Adjusted P-value	Representative Genes	Statistical Status
**GSE97760**	GO:BP	GO:0043161	Proteasome-mediated ubiquitin-dependent protein catabolic process	16	0.263	SMURF2, HACE1, UCHL5, UBQLN2, UBE4A	Top-ranked, not FDR-significant
**GSE97760**	GO:BP	GO:0010498	Proteasomal protein catabolic process	17	0.303	SMURF2, HACE1, UFL1, UCHL5, UBQLN2	Top-ranked, not FDR-significant
**GSE97760**	GO:BP	GO:0043484	Regulation of RNA splicing	8	0.439	HMX2, CLK4, SRSF1, RBM25, TIA1	Top-ranked, not FDR-significant
**GSE97760**	KEGG	hsa04120	Ubiquitin mediated proteolysis	6	0.822	SMURF2, UBE2E1, CUL5, BIRC3, UBE4A	Top-ranked, not FDR-significant
**GSE97760**	KEGG	hsa04141	Protein processing in endoplasmic reticulum	6	0.923	SVIP, EDEM3, SEC24A, BAG1, UBQLN2	Top-ranked, not FDR-significant
**GSE63060**	GO:BP	GO:0002181	Cytoplasmic translation	11	2.01 × 10^-11	RPS25, RPS24, RPL26, RPL21, RPS17	FDR-significant
**GSE63060**	GO:BP	GO:0019646	Aerobic electron transport chain	6	1.60 × 10^-5	NDUFA1, NDUFS5, UQCRH, UQCRHL, COX7C	FDR-significant
**GSE63060**	GO:BP	GO:0042773	ATP synthesis coupled	6	1.60 × 10^-5	NDUFA1, NDUFS5,	FDR-significant

			electron transport			UQCRH, UQCRHL, COX7C	
**GSE63060**	KEGG	hsa05012	Parkinson disease	9	3.00 × 10^-6	NDUFA1, NDUFS5, UQCRH, TXN, COX7C	FDR-significant *
**GSE63060**	KEGG	hsa00190	Oxidative phosphorylation	6	6.70 × 10^-5	NDUFA1, NDUFS5, UQCRH, UQCRHL, COX7C	FDR-significant

Note:
Top-ranked enrichment terms are shown for the two AD comparator cohorts. GSE97760 did not yield FDR-significant enrichment and is therefore summarized using the highest-ranked nominal trends, which were mainly related to proteostasis, ubiquitin-dependent proteolysis, and RNA-splicing regulation. In contrast, GSE63060 showed significant enrichment dominated by translation- and mitochondrial respiration-related processes. *The disease-named KEGG term "Parkinson disease" in GSE63060 was interpreted cautiously, as it was driven largely by broadly shared mitochondrial and respiratory-chain genes rather than taken as evidence of PD-specific recapitulation. Adjusted *P*-values were corrected using the Benjamini–Hochberg procedure. Count denotes the number of overlapping genes in each enriched term.

Abbreviations:
AD, Alzheimer's disease; BP, biological process; FDR, false discovery rate; GO, Gene Ontology; KEGG, Kyoto Encyclopedia of Genes and Genomes; PD, Parkinson's disease; UC, ulcerative colitis.

2 Supplementary Figures

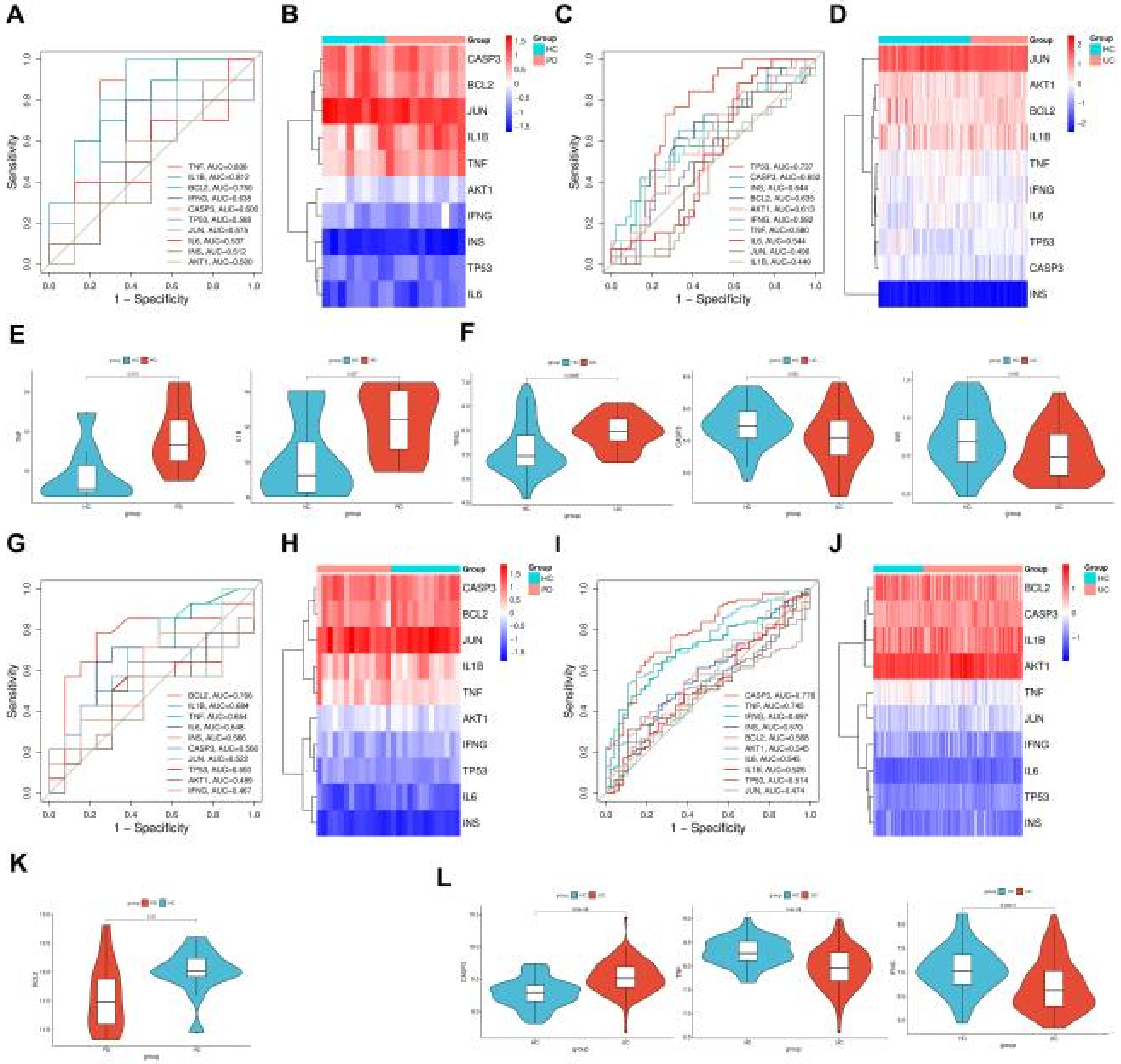

Supplementary Figure 1. Descriptive expression and external validation profiles of topology-derived core genes in PD and UC cohorts.

(A, C) Receiver operating characteristic (ROC) curves evaluating the discriminatory capacity of the ten core genes in the PD discovery cohort GSE22491 (A) and UC discovery cohort GSE3365 (C). The area under the curve (AUC) values provide descriptive, cohort-specific estimates of gene-level discriminatory performance rather than definitive biomarker validation. (B, D) Hierarchical clustering heatmaps of the ten core genes in PD (B) and UC (D) discovery cohorts. Samples are color-coded (red, disease; blue, healthy control). Clustering was performed using the complete linkage method with Euclidean distance to visualize coordinated expression patterns between groups. (E, F) Box plots showing differential expression of representative core genes in PD (E) and UC (F) discovery cohorts. Statistical significance was assessed by the two-tailed Wilcoxon rank-sum

test (*P < 0.05, **P < 0.01, ***P < 0.001*). Data are presented as median with interquartile range (IQR). (G, I) ROC curves for the ten topology-derived core genes in the independent PD cohort GSE75249 (G) and UC cohort GSE119600 (I), illustrating heterogeneous external performance rather than uniform robustness. (H, J) Hierarchical clustering heatmaps of the ten core genes in the PD validation cohort (H) and UC validation cohort (J). (K, L) Violin plots illustrating expression levels of core genes that maintained significant differential expression in the PD validation cohort GSE75249 (K) and UC validation cohort GSE119600 (L). Each dot represents an individual sample. In the external cohorts, BCL2 showed the clearest signal in PD, whereas *CASP3, TNF,* and *IFNG* were relatively more reproducible in UC. Statistical significance was assessed by the two-tailed Wilcoxon rank-sum test (*P < 0.05, **P < 0.01, ***P < 0.001*). Data are presented as median with IQR.

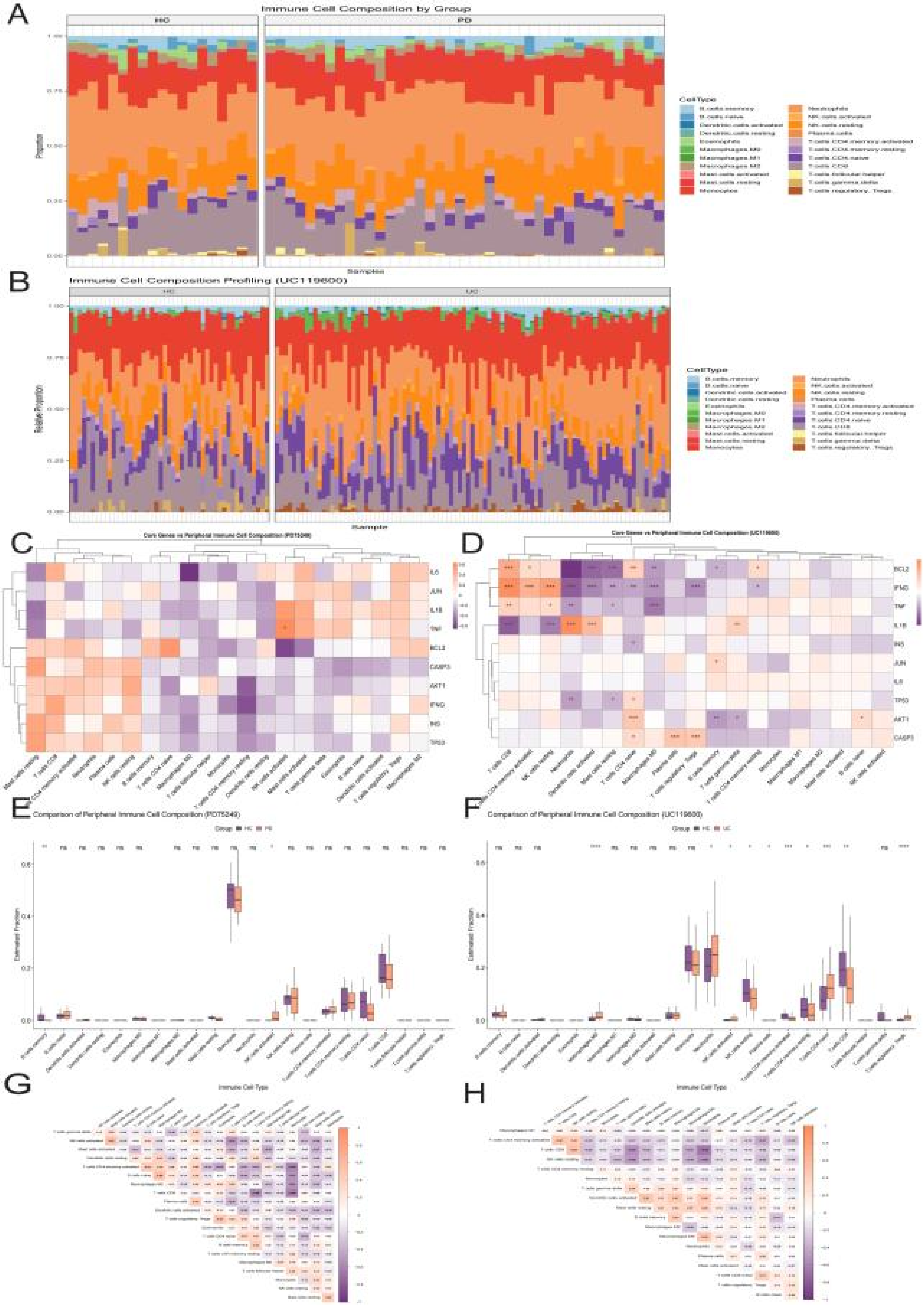
A
Immune Cell Composition by Group
HC
PD
Samples
CellType
B
Immune Cell Composition Profiling (UC119600)
HC
UC
Sample
CellType
C
D
E
F
G
Immune Cell Type
H
Immune Cell Type

Supplementary Figure 2. Independent immune-cell validation in PD and UC cohorts.

(A–B) Relative immune cell proportions in validation cohorts. Stacked bar plots showing the estimated fractions of 22 leukocyte subsets (LM22) in the PD validation cohort GSE75249 (A) and UC validation cohort GSE119600 (B). These profiles illustrate the heterogeneity of the peripheral immune microenvironment across independent patient samples. (C–D) Correlation between core genes and immune cell subsets. Spearman correlation heatmaps depict the relationships between the ten core genes and immune cell fractions in the PD (C) and UC (D) validation cohorts. Partially conserved correlation patterns across datasets support a reproducible but not identical inflammatory context in both disorders. (E–F) Group-level comparisons of immune cell composition. Box plots show differential immune cell fractions between patients and healthy controls (HC) in the PD (E) and UC (F) validation cohorts. Significant alterations were observed in selected cell types, including memory B cells, activated NK cells, macrophages M0, and regulatory T cells. Statistical significance was assessed by the Wilcoxon rank-sum test ($*P < 0.05$, $**P < 0.01$, $***P < 0.001$). (G–H) Inter-cell correlation networks. Correlation matrices of the 22 immune cell types in the PD (G) and UC (H) validation cohorts. All P-values were adjusted for multiple comparisons using the Benjamini–Hochberg method.

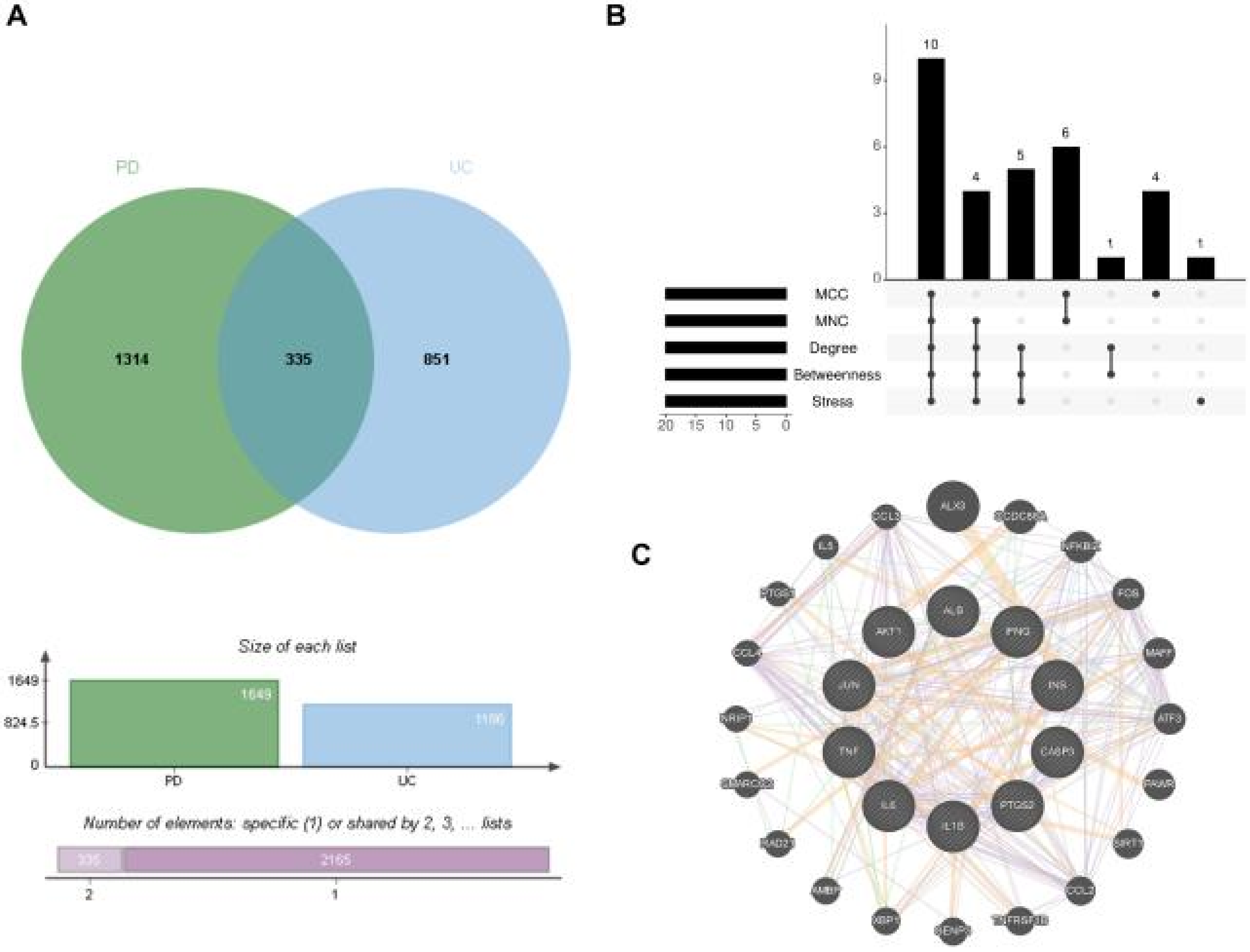

Supplementary Figure 3. Sensitivity analysis and functional characterization of shared Parkinson's disease and ulcerative colitis signatures based on relaxed statistical thresholds.

(A) Identification of shared signatures. Venn diagram showing the intersection of disease-associated genes from multiple sources. Shared signature genes were identified by integrating transcriptomic differentially expressed genes (DEGs) from GEO discovery datasets (GSE22491 for PD and GSE3365 for UC; threshold: adjusted $P < 0.05$ and $|\log2FC| > 1$) with curated disease-associated genes from GeneCards, DisGeNET, and CTD databases. The resulting overlap represents the shared gene set under conventional significance criteria. (B) Consensus core gene selection. UpSet plot visualizing the robust identification of the ten core genes (*TNF, IL1B, TP53, AKT1, CASP3, IL6, BCL2, IFNG, INS, and JUN)*. These genes were consistently ranked among the top candidates across five complementary topological algorithms (Degree, MCC, MNC, Stress, and Betweenness) using the CytoHubba plugin, demonstrating that the core hub architecture remains stable even under relaxed statistical thresholds. (C) Functional interaction network of core genes. GeneMANIA network illustrating the biological interplay among the ten core genes. The network integrates multiple lines of evidence, primarily co-expression (71.05%) and predicted functional associations (18.15%), supporting the stability of the core network topology under relaxed DEG thresholds.

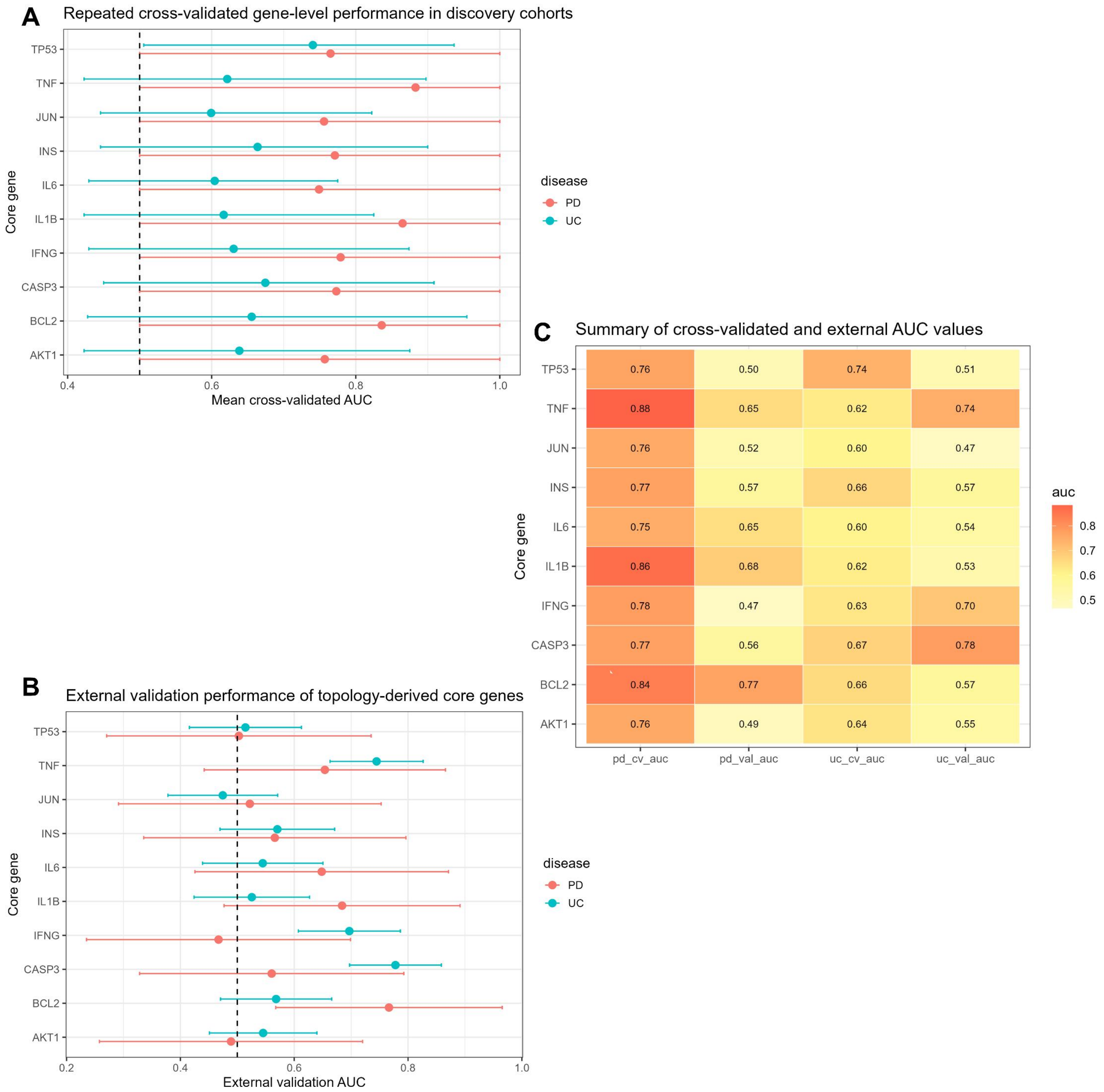

Supplementary Figure 4. Cross-validated and external validation assessment of topology-derived core genes in Parkinson's disease and ulcerative colitis cohorts.

(A) Repeated stratified 5-fold cross-validated area under the curve (AUC) values for each topology-derived core gene in the discovery cohorts of Parkinson's disease (PD) and ulcerative colitis (UC). Points indicate mean AUC values across repeated iterations, and error bars indicate empirical 95% intervals derived from the cross-validation procedure. A dashed horizontal line marks the chance level (AUC = 0.5).
(B) External validation AUC values for each topology-derived core gene in the corresponding independent PD and UC validation cohorts. Points indicate AUC values, and error bars indicate 95% confidence intervals. A dashed horizontal line marks the chance level (AUC = 0.5).
(C) Heatmap-style summary of cross-validated and external validation AUC values for all topology-derived core genes across the PD and UC analysis framework. Numeric values are shown within each tile to facilitate

direct comparison.
These analyses were performed separately in PD and UC to assess gene-level stability and reproducibility across cohorts. The results showed heterogeneous discriminatory performance among the topology-derived core genes, with *TNF, CASP3, and BCL2* showing relatively more stable performance, whereas several other genes displayed context-dependent or weak standalone discriminatory ability.

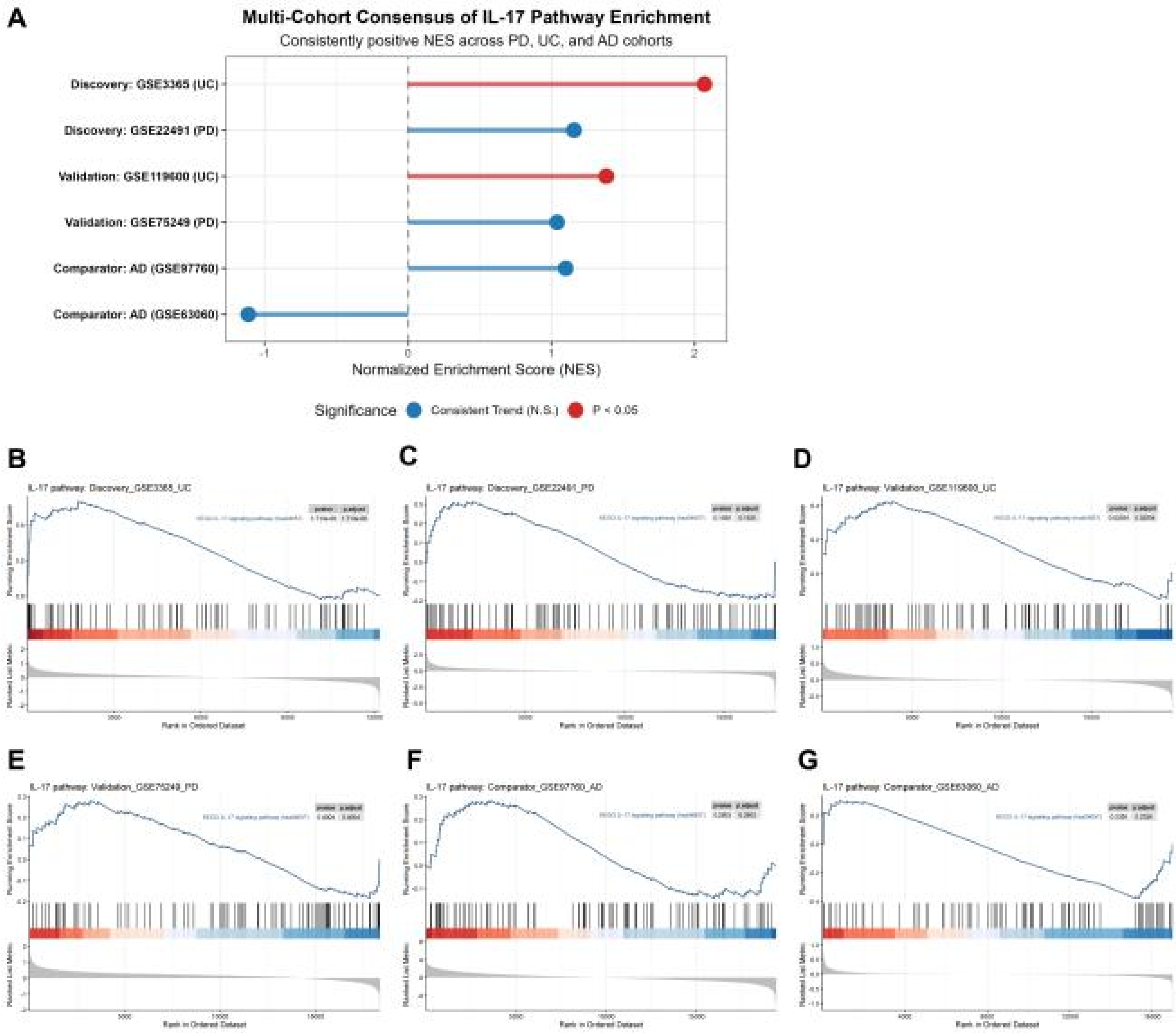

Supplementary Figure 5. Independent cohort-level evaluation of IL-17 signaling pathway enrichment across discovery, validation, and comparator datasets

(A) Summary plot of normalized enrichment scores (NES) for the KEGG **IL-17 signaling pathway** (**hsa04657**) across the discovery, validation, and comparator cohorts, including **GSE3365** (UC discovery), **GSE22491** (PD

discovery), **GSE75249** (PD validation), **GSE119600** (UC validation), **GSE97760** (AD comparator), and **GSE63060** (AD comparator). Colors indicate whether pathway enrichment reached statistical significance in the corresponding dataset.

(B–G) Gene set enrichment analysis (GSEA) plots for the IL-17 signaling pathway in individual cohorts: (B) GSE3365, (C) GSE22491, (D) GSE119600, (E) GSE75249, (F) GSE97760, and (G) GSE63060. Genes were ranked according to differential expression in disease versus control samples, and preranked GSEA was performed using the KEGG IL-17 signaling pathway gene set. Positive NES values indicate relative enrichment of IL-17-related genes toward the upregulated end of the ranked gene list. Significant enrichment was observed in the UC cohorts, whereas the PD cohorts were positive but non-significant and the AD comparator results were non-significant and directionally inconsistent.

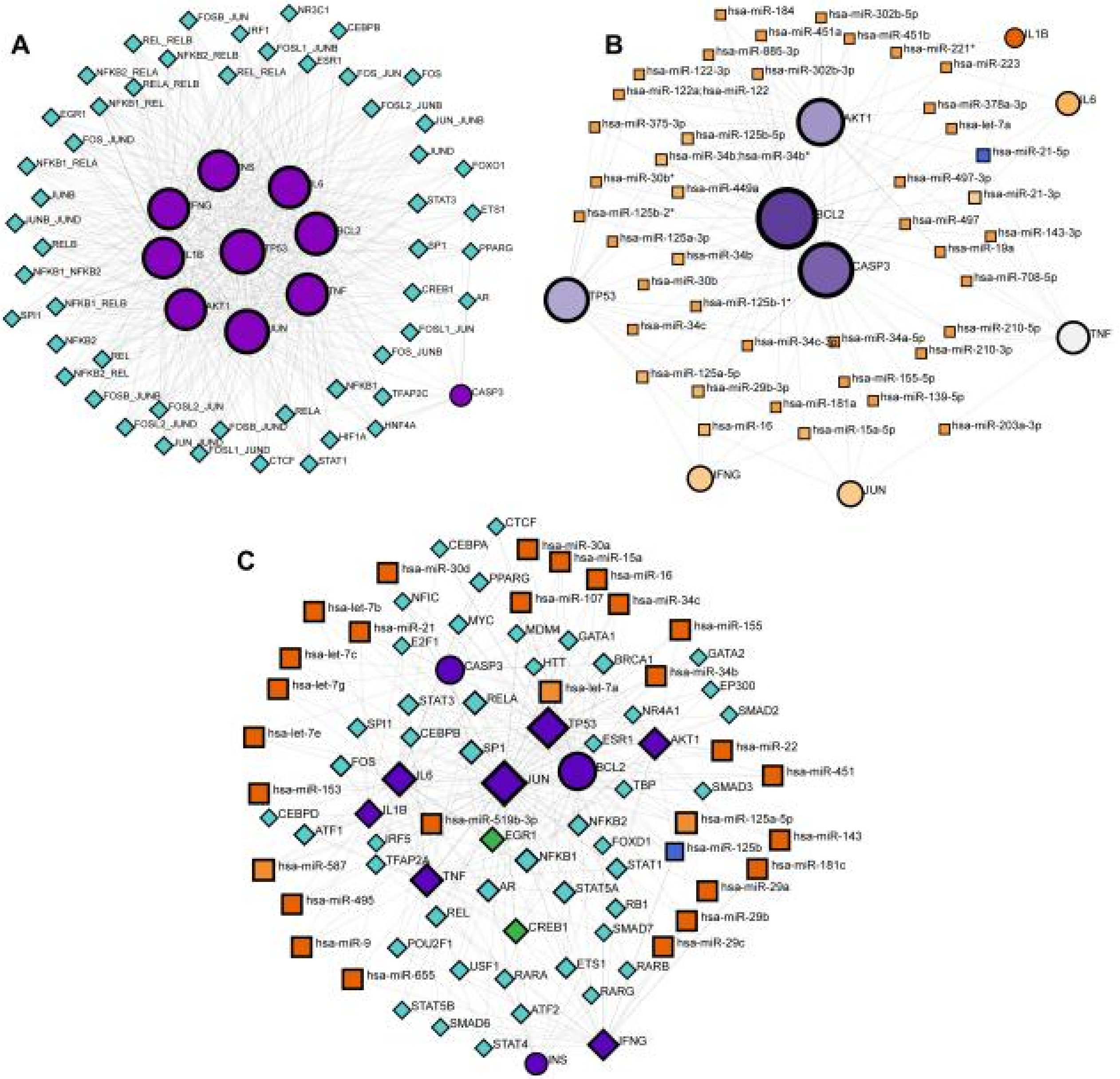

Supplementary Figure 6. Transcriptional and post-transcriptional regulatory architecture of PD–UC core genes.

Transcription factor (TF)–gene interaction network. Squares represent upstream TFs and circles represent the identified core genes as predicted via the OmniPath database. TP53 and JUN emerge as the primary regulatory hubs coordinating the transcriptional landscape across PD and UC. (B) MicroRNA (miRNA)–gene interaction network. Integrated mapping of miRNAs targeting the ten core genes. hsa-miR-21-5p demonstrates significant regulatory breadth, suggesting its role as a potential post-transcriptional modulator within the gut-brain axis. (C) Integrated TF–miRNA regulatory circuit. A synergistic regulatory landscape of the core genes was established using RegNetwork to illustrate coordinated control at both transcriptional and post-transcriptional levels. All networks were filtered using a degree-based strategy (Degree > 7 for TFs, Degree > 2 for miRNAs, and Degree > 1 for integrated circuits) to prioritize high-confidence regulatory interactions and minimize topological noise.

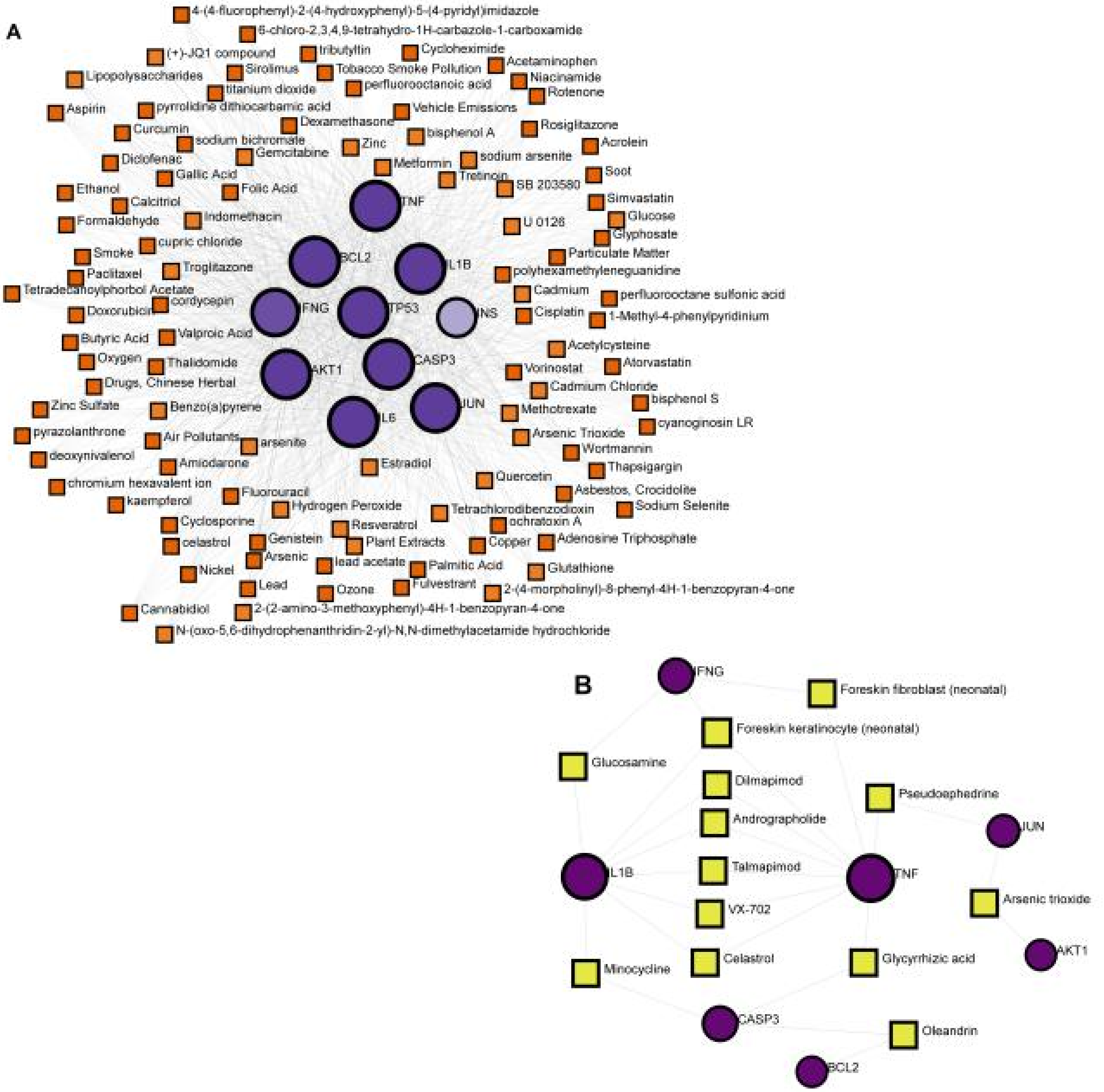

Supplementary Figure 7. Integrative analysis of protein–chemical and protein–drug interaction networks.

(A) Protein–chemical interaction network. Visualization of the interplay between the ten core proteins (circles) and bioactive chemical compounds (squares) retrieved from the Comparative Toxicogenomics Database (CTD). This network identifies key nodes, such as CASP3 and TNF, that integrate responses to environmental stressors and bioactive compounds, highlighting the susceptibility of the PD–UC axis to exogenous modulation. (B) Protein–drug interaction network. Mapping of therapeutic agents (squares) targeting the core proteins (circles) based on the DrugBank database. The network identifies several candidate compounds for future experimental evaluation rather than direct evidence of therapeutic efficacy in PD–UC

comorbidity. All nodes were prioritized using an adaptive topological threshold (Degree > 8 for chemicals; Degree > 1 for drugs) to ensure the identification of functionally relevant therapeutic hubs.

3 Supplementary Data

The Supplementary Data supporting the findings of this study are available in the Figshare repository under the following DOI upon publication: 10.6084/m9.figshare.31423433. For preview during the review process, the data can be accessed via the private sharing link: https://figshare.com/s/7fe48ed5f803c0ea5ee9.

Supplementary Data 1. Differential Expression Gene Lists

Complete lists of differentially expressed genes (DEGs) identified in the discovery cohorts (GSE22491 for Parkinson's disease, GSE3365 for ulcerative colitis) and the two Alzheimer's disease comparator cohorts (GSE97760 and GSE63060). Each cohort's DEG results are provided in a separate sheet, including gene symbols, log2 fold change, adjusted P-values, and other relevant statistics.

Supplementary Data 2. Shared Signature Genes (320 Genes) and Evidence Sources

Detailed inventory of the 320 genes commonly associated with Parkinson's disease and ulcerative colitis. The file contains one main sheet listing all genes, along with columns indicating their source databases (GeneCards, CTD, DisGeNET, GEO differential expression) for both diseases. Cross- referencing of evidence supports the identification of robust shared signatures.

Supplementary Data 3. Functional Enrichment Results (GO/KEGG)

Comprehensive enrichment analysis output for four analytical contexts: the 320 shared genes, the 10 core hub genes, and the two Alzheimer's disease comparator cohorts. Separate sheets present Gene Ontology (Biological Process, Cellular Component, Molecular Function) and KEGG pathway terms, including gene counts, enrichment ratios, P-values, and false discovery rates.

Supplementary Data 4. Immune Infiltration Profiles (CIBERSORTx)

Complete results of peripheral immune cell deconvolution using CIBERSORTx. The Excel file includes the following sheets:

Cell Proportions Barplot: Estimated fractions of 22 immune cell subsets for each sample in discovery and validation cohorts.

Immune Abundance Boxplot: Comparison of immune cell fractions between disease and control groups, with statistical significance.

Gene–Cell Correlation: Spearman correlation coefficients between core genes and immune cell subsets.

Cell–Cell Interactions: Correlation matrices depicting synergistic/antagonistic relationships among immune cell types.

Supplementary Data 5. Regulatory Networks and Therapeutic Candidates

Sheet Name	Content Description	Database / Source	Screening Criteria (Threshold)
TFs	Transcription factors targeting core genes	OmniPath	Degree > 7, Betweenness > 0
miRNAs	microRNAs targeting core genes	OmniPath	Degree > 2, Betweenness > 0
TF-miRNA	Integrated regulatory loops (TF-miRNA coregulation)	RegNetwork	Degree > 1, Betweenness > 0
Chemicals	Chemicals interacting with core proteins	CTD Database	Degree > 8, Betweenness > 0
Drugs	Potential therapeutic drugs targeting core proteins	DrugBank 5.0	Degree > 1, Betweenness > 0
